# Computational tools for the prediction of site- and regioselectivity of organic reactions

**DOI:** 10.1039/d5sc00541h

**Published:** 2025-03-04

**Authors:** Lukas M. Sigmund, Michele Assante, Magnus J. Johansson, Per-Ola Norrby, Kjell Jorner, Mikhail Kabeshov

**Affiliations:** a Molecular AI, Discovery Sciences, R&D, AstraZeneca Gothenburg Pepparedsleden 1 43183 Mölndal Sweden lukas.sigmund@astrazeneca.com mikhail.kabeshov@astrazeneca.com; b Innovation Centre in Digital Molecular Technologies, Department of Chemistry, University of Cambridge Lensfield Rd Cambridge CB2 1EW UK; c Compound Synthesis & Management, The Discovery Centre, AstraZeneca Cambridge Cambridge Biomedical Campus, 1 Francis Crick Avenue CB2 0AA Cambridge UK; d Medicinal Chemistry, Research and Early Development, Cardiovascular, Renal and Metabolism (CVRM), BioPharmaceuticals, R&D, AstraZeneca Gothenburg Pepparedsleden 1 43183 Mölndal Sweden; e Data Science & Modelling, Pharmaceutical Sciences, R&D, AstraZeneca Gothenburg Pepparedsleden 1 43183 Mölndal Sweden; f ETH Zürich, Institute of Chemical and Bioengineering, Department of Chemistry and Applied Biosciences Vladimir-Prelog-Weg 1 CH-8093 Zürich Switzerland; g National Centre of Competence in Research (NCCR) Catalysis, ETH Zurich Zurich Switzerland kjell.jorner@chem.ethz.ch

## Abstract

The regio- and site-selectivity of organic reactions is one of the most important aspects when it comes to synthesis planning. Due to that, massive research efforts were invested into computational models for regio- and site-selectivity prediction, and the introduction of machine learning to the chemical sciences within the past decade has added a whole new dimension to these endeavors. This review article walks through the currently available predictive tools for regio- and site-selectivity with a particular focus on machine learning models while being organized along the individual reaction classes of organic chemistry. Respective featurization techniques and model architectures are described and compared to each other; applications of the tools to critical real-world examples are highlighted. This paper aims to serve as an overview of the field's *status quo* for both the intended users of the tools, that is synthetic chemists, as well as for developers to find potential new research avenues.

## Abstract 2

This article reviews computational tools for the prediction of the regio- and site-selectivity of organic reactions. It spans from quantum chemical procedures to deep learning models and showcases the application of the presented tools.

## Graphical abstract

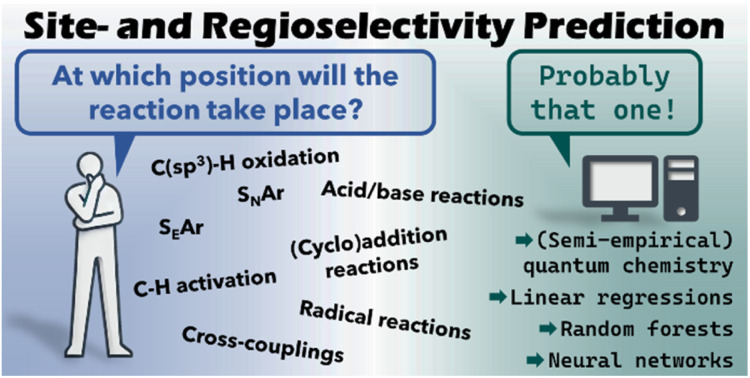

## Introduction

Organic synthesis deals with the design and production of new molecules. En route to this objective, numerous considerations need to be taken into account, and their sum influences the overall outcome of any synthesis. Besides the CO_2_ footprint and sustainability in general, the key aspects are the yield of the desired product as well as the by-product profile including possible isomers. Therefore, the prediction of reaction feasibility and selectivity is of paramount importance for the planning of organic syntheses. Today more than ever, computers are indispensable assistants for researchers to tackle these challenges, not least due to the massive increase in the amount of available data through high throughput experimentation (HTE) and synthesis automation.^1–5^ Models built on this data can assist in predicting individual parameters and as a result, reduce attrition in synthesis efforts in areas like medicinal or agricultural chemistry as well as materials science.

In the last ten years, machine learning (ML) has tremendously changed the field of chemical synthesis prediction by processing massive amounts of either experimentally obtained or computationally generated data into predictive tools.^6–10^ This created a plethora of new research opportunities but also challenges in the field of organic synthesis, including the need to produce data suitable for data science. Navigating this new landscape is the current task of the scientific community and warrants the close collaboration of model developers and users, that is synthetic chemists, to leverage ML to its full potential.^11^

In this review article, we focus on predictive digital tools for regio- and site-selectivity – a long-standing research field in computational organic chemistry. Methods for the prediction of stereoselectivity are beyond the scope of this paper. Several review and perspective articles have been published on this topic.^12–19^ Also, the closely related field of chemoselectivity is not discussed in detail and is only briefly mentioned where appropriate.^20^

**Fig. 1:**
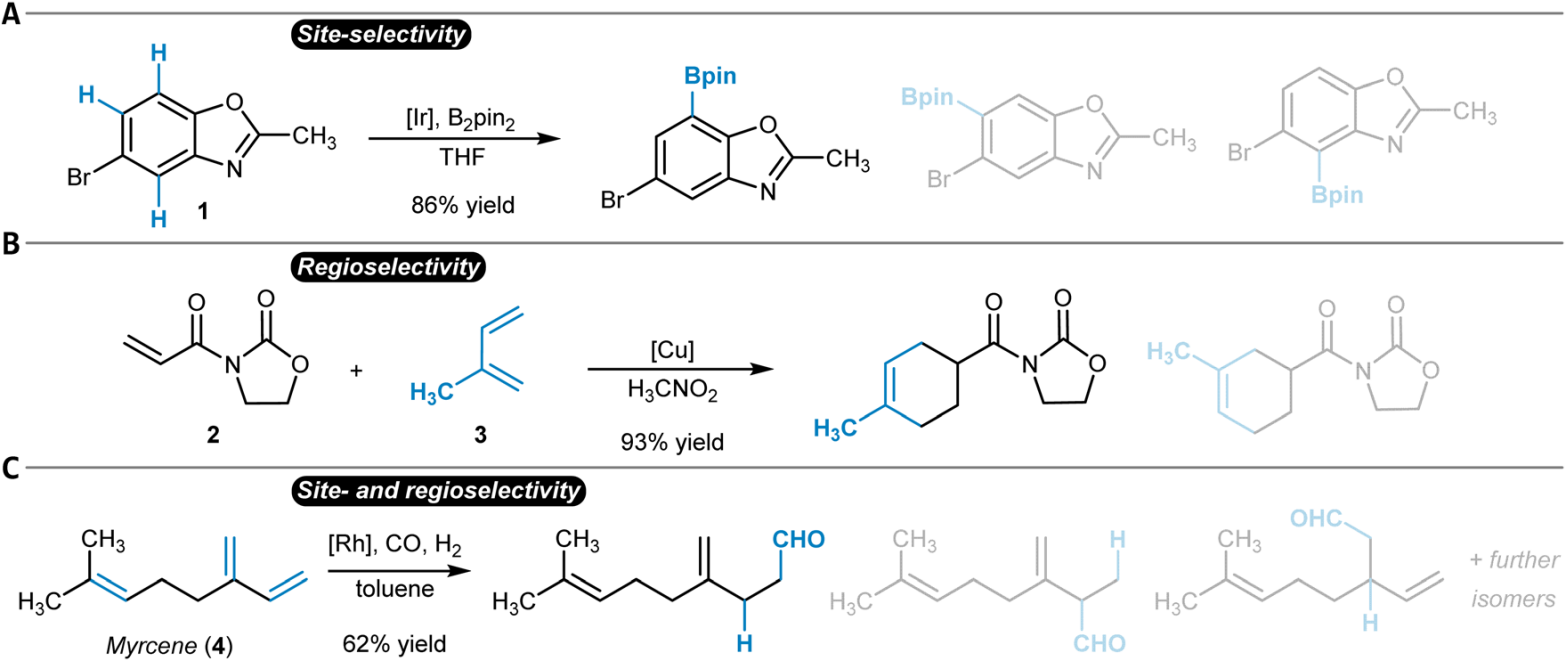
Site- and regioselectivity of organic reactions. (A) Iridium-catalyzed site-selective borylation that proceeds primarily at one of the three possible C_aromatic_–H groups. (B) Copper-catalyzed regioselective Diels–Alder reaction. (C) Rhodium-catalyzed hydroformylation of myrcene with high site- and regioselectivity. In all cases, the main reaction product is shown first, after which additional possible isomers are given half-transparently.

While the terms regioselectivity and site-selectivity are often used synonymously, they can serve to describe slightly different observations. We herein make use of this distinction and would like to illustrate it with three examples. Heteroarene 1 is borylated with high site-selectivity while the reaction does not bring a regioselectivity question (Fig. 1A).^21^ Site-selectivity refers to a reaction that takes place at a clearly defined position of a substrate molecule (*e.g.*, a C_aromatic_–H group) among several other identical options (sites). Complementarily, the Diels–Alder reaction between dienophile 2 and diene 3 proceeds with high regioselectivity without the possibility of site-isomeric products (Fig. 1B).^22^ This is due to the preferential orientation of the two reactants relative to each other during the bond-forming process.^23,24^ More complicated is for instance the hydroformylation of myrcene (4), which can result in a diverse mixture of reaction products due to potentially low site- and regioselectivity (Fig. 1C).^25^ A discussion of the underlying physical principles of selectivity in chemistry is provided below at the end of the section on general reactivity models for site- and regioselectivity prediction.

This paper is structured as follows: initially, molecular featurization techniques, as well as (ML) model architectures used for site- and regioselectivity prediction, are presented briefly. Next, general reaction prediction models are discussed with respect to regio- and site-selectivity. The successive four sections deal with models for C(sp^3^)–H, C(sp^2^)–H, C(sp^2^)–X, as well as double and triple-bond functionalization reactions. The final part of the paper makes concluding remarks and takes a view to potential future developments. It also includes with Table 1 a summary of important computational tools reviewed herein with direct web links to respective GitHub repositories or online graphical user interfaces. This gives a straightforward overview of the available models and enables easy access to them.

**Table 1:** Overview of computational tools and associated resources for site- and regioselectivity prediction. All given links were successfully accessed in January 2025

	Name, reaction type, and reference	Model type	Web links
1	**Molecular transformer**: general reaction prediction tool^101^	Transformer	https://github.com/pschwllr/MolecularTransformer and https://rxn.app.accelerate.science/rxn/sign-in
2	**pKalculator**: C–H deprotonation^154^	SQM and LightGBM	https://github.com/jensengroup/pKalculator and https://regioselect.org/
3	**RegioSQM**: S_E_Ar^84,192^	SQM	http://regiosqm.org/, https://regioselect.org/, and https://github.com/jensengroup/RegioSQM20
4	S_E_Ar^198^	RF	https://github.com/Ianiusha/AutoLSF/tree/master/EAS
5	**RegioML**: S_E_Ar^199^	LightGBM	https://github.com/jensengroup/RegioML
6	C_aromatic_–H functionalization^201^	GNN	https://askcos.mit.edu/forward?tab=sites
7	**ml-QM-GNN**: primarily aromatic substitution^62^	GNN	https://github.com/yanfeiguan/reactivity_predictions_substitution
8	Radical C_aromatic_–H substitution^220^	RF	https://github.com/Masker-Li/ChemSelML
9	Minisci-type functionalizations^222^	GNN	https://github.com/emmaking-smith/SET_LSF_CODE
10	Pd-catalyzed C_aromatic_–H activation^234^	DFT	https://github.com/sustainable-processes/Pd-catalysed_C-H_activation_reaction_prediction
11	Electrocatalyzed arene alkenylation^236^	Extra trees	https://zenodo.org/records/8003927
12	**RegioTM**: Pd-catalyzed C_aromatic_–H activation^241^	SQM	https://github.com/jensengroup/regiotm
13	**SoBo**: Ir-catalyzed C_aromatic_–H borylation^250^	SQM + PLS and MLR	https://github.com/C-H-activation/ICB-workflow and https://pypi.org/project/sobo/
14	C–H borylation^251^	GNN	https://github.com/ETHmodlab/lsfml
15	Ir-catalyzed C_aromatic_–H borylation^255^	Transformer	https://github.com/ruslankotl/rxn-data-proc
16	**Regio-MPNN**: cross-coupling^289^	GNN	https://ai.tools.chemlex.com/region-choose and https://github.com/Chemlex-AI/regioselectivity
17	S_N_Ar^301^	Gaussian process	https://pubs.rsc.org/en/content/articlelanding/2021/sc/d0sc04896h
18	S_N_Ar^302^	GNN + DFT	https://pubs.acs.org/doi/10.1021/acs.jcim.3c00580
19	**HeckQM**: Mizoroki–Heck reaction^242^	SQM + DFT	https://github.com/jensengroup/HeckQM
20	Hydroformylation^320^	XGBoost	https://github.com/3xbs3/Hydroformylation
21	Diels–Alder reaction^346^	GNN	https://github.com/angusketo/DA_DataExtraction

## Molecular representations and featurization

**Fig. 2:**
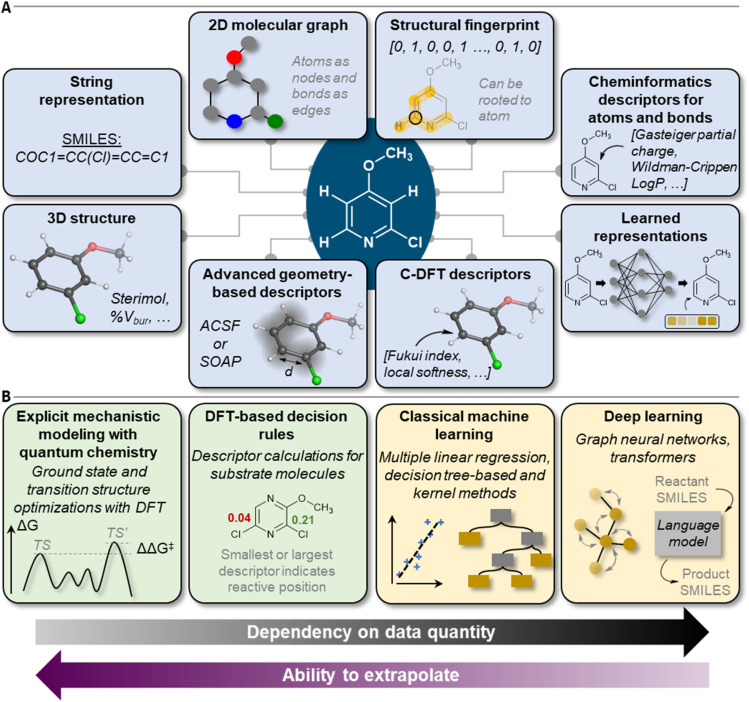
Overview of (A) molecular features, descriptors, and representations and (B) model types for site- and regioselectivity prediction.

Many approaches have been developed to incorporate molecular information into machine-readable format (Fig. 2A).^26–28^ In terms of site- and regioselectivity prediction, the chosen featurization technique must allow for the local description of a given position of a molecule instead of characterizing it as one entity.^29^ In addition to local information, global information can also be of relevance, for example, to address questions on reaction feasibility or selectivity between competing sites. The compute time to obtain expressive position-specific features is another important aspect, especially when it comes to the application of the models by the users. Faster methods with rather low computational resource demand can be deployed more broadly, also to large compound libraries, and without the mandatory need for high-performance computer resources. However, the lower computational cost must be balanced with the potentially lower generalizability of the chosen featurization procedure and its accuracy in combination with the trained predictive model.

The most common string representation of a molecule with regard to computational modeling is the Simplified Molecular Input Line Entry System (SMILES).^30^ SMILES strings can be supplemented with atom mapping numbers or wildcard atoms which can be used to identify or mark certain positions within a molecule or molecular fragment. Likewise, SMILES strings can be further processed with cheminformatics software like RDKit^31^ to generate alternative molecular representations; for instance, two-dimensional molecular graphs with atoms as nodes and bonds as edges. Graphs inherently give access to specific sites of molecules due to varying node and edge attributes. Also, substructure matching can be applied to locate defined regions. Cheminformatics fingerprints encode molecules in bit vectors and are calculated from molecular graphs.^32^ They can be rooted to atoms to generate position-specific data in addition to global fingerprints. There is also a variety of atom-centered cheminformatics descriptors such as Gasteiger–Marsili partial charges,^33^ Wildman–Crippen indices,^34^ or eigenvalues of Burden matrices.^35^ They all can be rapidly calculated and do not require a three-dimensional molecular structure.

At the same time, significant progress was achieved in generating three-dimensional representations from two-dimensional molecular graphs.^36,37^ For organic molecules, this is now possible with a high degree of reliability. For metal-containing structures and inorganic molecules in general, this is more challenging; however, there are dedicated and quite robust implementations available.^38^ Three-dimensional molecular structures allow to capture effects like intramolecular interactions or steric influences more accurately, which can influence site-specific reactivity. They grant access to many further local features like atomic distances, relative buried volumes,^39–41^ or Sterimol parameters.^42,43^ More sophisticated geometrically-inspired local descriptors are atom-centered symmetry functions (ACSF)^44^ or smooth overlap of atomic positions (SOAP).^45^ However, with three-dimensional molecular representations, new challenges like the navigation of conformational ensembles or the computational level of structural optimization^46^ need to be addressed, which influences feature values, model training, and execution times significantly.^47–49^

Three-dimensional molecular information (commonly in the form of *xyz* coordinates) also serves as input to quantum chemical simulation software. Outstanding is the area of conceptual density functional theory (C-DFT)^50–52^ which has produced several atom-specific descriptors of high regio- and site-predictive power. Prominent examples are the condensed Fukui indices^53,54^ which quantify the redistribution of electron density for each atom upon electron removal or addition to a given molecule. Most commonly, an entire electron is added or removed, and the indices are typically calculated as differences in atomic partial charges to indicate nucleophilic and electrophilic properties, respectively. The Fukui functions can also be approximated with spin densities^55–57^ and by frontier molecular orbital theory,^57,58^ respectively, and related reactivity descriptors have been derived, too.^59,60^ The benefit of the resulting data is the high degree of generalizability due to being strongly rooted in quantum mechanics (QM), while the downsides are the high demands on computational resources and time.

A remedy for the dilemma between accuracy and compute time/power is provided, for example, by parametrized semi-empirical versions of density functional theory (DFT) such as tight-binding DFT.^61^ They offer the descriptors at a significantly reduced time and hardware cost. Another promising approach that has been pursued lately is the training of ML regressors of DFT descriptors.^62^ These models are orders of magnitude faster than the physics-based simulations while still providing sufficient levels of accuracy. The ML DFT descriptors can then be interpreted directly or used within a separate model for regio- or site-selectivity prediction.^63^ Careful consideration of the applicability domain of the ML regressor models is required when using these techniques. Nevertheless, the combination of fast quantum chemical calculations with statistical methods is a promising approach that will be discussed multiple times throughout this review paper.

Ultimately, the above-mentioned molecular representations can be used with deep ML models to learn improved representations from the initial input features during a predefined learning exercise. A common scenario is that of a graph neural network (GNN) trained with molecular graphs annotated with simple node and edge features such as atom or bond type.^64^ During training, atom and bond-centered embedding vectors are learned, which can be used for regio- and site-selectivity prediction. An exciting development is using quantum chemical descriptors as input features in GNNs in addition to the conventional atom, bond, and molecular features.^62,63,65^ It has been shown that the additional information from the QM descriptors is helpful for prediction tasks with less than around 2000 datapoints.^65^ Approaches based on three-dimensional electronic density grids and the spherical steric environment centered on each atom have also been pursued.^66^

Numerous software packages have been developed for the generation of site-agnostic features for ML models. These include for instance cheminformatics tools like RDKit,^31^ kallisto,^67^ DBStep,^68^ SambVca,^40^ morfeus,^69^ DScribe,^70^ or others.^71^ Typical quantum chemical software like Gaussian^72^ or ORCA,^73^ or semi-empirical quantum mechanics (SQM) implementations like xtb^74^ can also be used to calculate features, optionally combined with further analyses of the electronic structure, for example, with Multiwfn.^75^

## Models

With the advent of practical and sufficiently fast computational chemistry methods in the 1970's, reaction mechanisms could be interrogated by simulations (Fig. 2B). While simulations were carried out manually for a long time, there are nowadays automated computational chemistry workflows that can calculate reaction paths including relative activation energies between competing reactions.^76–80^ Even though such workflows have been applied for the prediction of regio- and site-selectivity (see below), they come at a quite high computational cost and generally suffer from a lack of robustness in transition state optimizations (typically 50–80% success rate).^81–83^ Even though simulation workflows can be accelerated by using SQM methods rather than DFT,^84^ and in the future plausibly by reactive ML potentials,^85^ ML predictions represent an attractive alternative when training data is available.

In ML, computational algorithms are trained to obtain statistical models based on a given dataset. The resulting tools can then be applied to make predictions on new data. For regio- or site-selectivity prediction, a supervised learning strategy is typically followed, which relates a set of input features, that is molecular representations (see previous section) and potentially information on reaction conditions, to a target quantity. These target labels can either be categorical (classification task), for instance, defining a site of a molecule as reactive or unreactive, or continuous (regression task), for example, relative Gibbs free activation energies (ΔΔ*G*^‡^ models). Regression is more rigorous but also requires more detailed data, that is, relative amounts of regio- or site-isomeric products.

ML algorithms can be divided into the classical and the deep learning approaches (Fig. 2B). Algorithm selection is a multifaceted question that must take into account the size, quality, and composition of the dataset, the featurization technique that is applied to the molecules, and the degree of interpretability the trained model is expected to have. Also, aspects like overall model training and inference time in relation to available hardware capabilities should be considered.

The classical methods include a family of linear algorithms, for example (multiple) linear or logistic regression, and also a collection of kernel-based methods such as support vector machines or Gaussian processes.^86^ The tree-based algorithms like random forest (RF) and related approaches such as gradient-boosted decision trees might also be considered classical.^87,88^ Generally, these methods can be applied to rather small to medium-sized datasets (less than ≈500 datapoints),^89,90^ while often being suitable for large datasets, too, and are more straightforward to interpret in most cases.

Deep ML algorithms are built with artificial neural networks. Depending on the exact architecture, they can process a broad variety of different features that get transformed into learned representations during training. Illustrative is the case of GNNs, which operate with molecular graph inputs to learn atom, chemical bond, and molecular representations.^64^ The transformer architecture^91^ is another famous example of deep learning for natural language, which has seen its applications in chemistry (using, *e.g.*, the SMILES representation) and also more specifically for regio- and site-selectivity questions. Deep ML algorithms are generally more data-hungry, though approaches like transfer learning^92^ are used to counteract this limitation, and active learning^93^ can be applied to design datasets more effectively.

## General reactivity models for site- and regioselectivity prediction

The retrosynthetic analysis of a desired target molecule is one of the most sophisticated tasks in organic chemistry and as such also defined the entry point of computer programs into the science of chemical synthesis planning.^94–96^ Even today, the computational generation of retrosynthetic pathways (backward synthesis prediction) is an active research area with many open challenges and questions.^97^

At the same time, many computational tools for the prediction of chemical reaction outcomes in a general sense (forward synthesis prediction) have been developed.^6–10^ Early examples such as CAMEO^98^ rely on the implementation of human-derived and mechanistically motivated rules by recognizing reactive group templates. Later, template-free methods were developed representing molecules through graphs or SMILES strings used with deep learning models such as GNNs^64^ or language models.^99^

Both, the backward and forward synthesis planning tools come with a remarkable degree of generalizability which enables their application to a broad set of organic synthesis problems. However, this comes potentially at the cost of lower precision for more specific tasks such as site- or regioselectivity prediction. The tools are most often evaluated on a representative held-out test set of synthetic pathways or individual reaction steps as an attempt to indicate the models' general prediction ability. Dedicated analyses of more specific tasks are not so common. Nevertheless, it can be argued that a general synthesis prediction software must solve regio- and site-selectivity questions implicitly^100^ by learning from large datasets that are assumed to contain sufficient information to accomplish that. However, both the availability of high-quality data as well as the models' capability to learn intrinsically complex chemistry knowledge can be limited. Hence, insight into the general models' actual predictive power in the context of regio- and site-selectivity is needed.

**Fig. 3:**
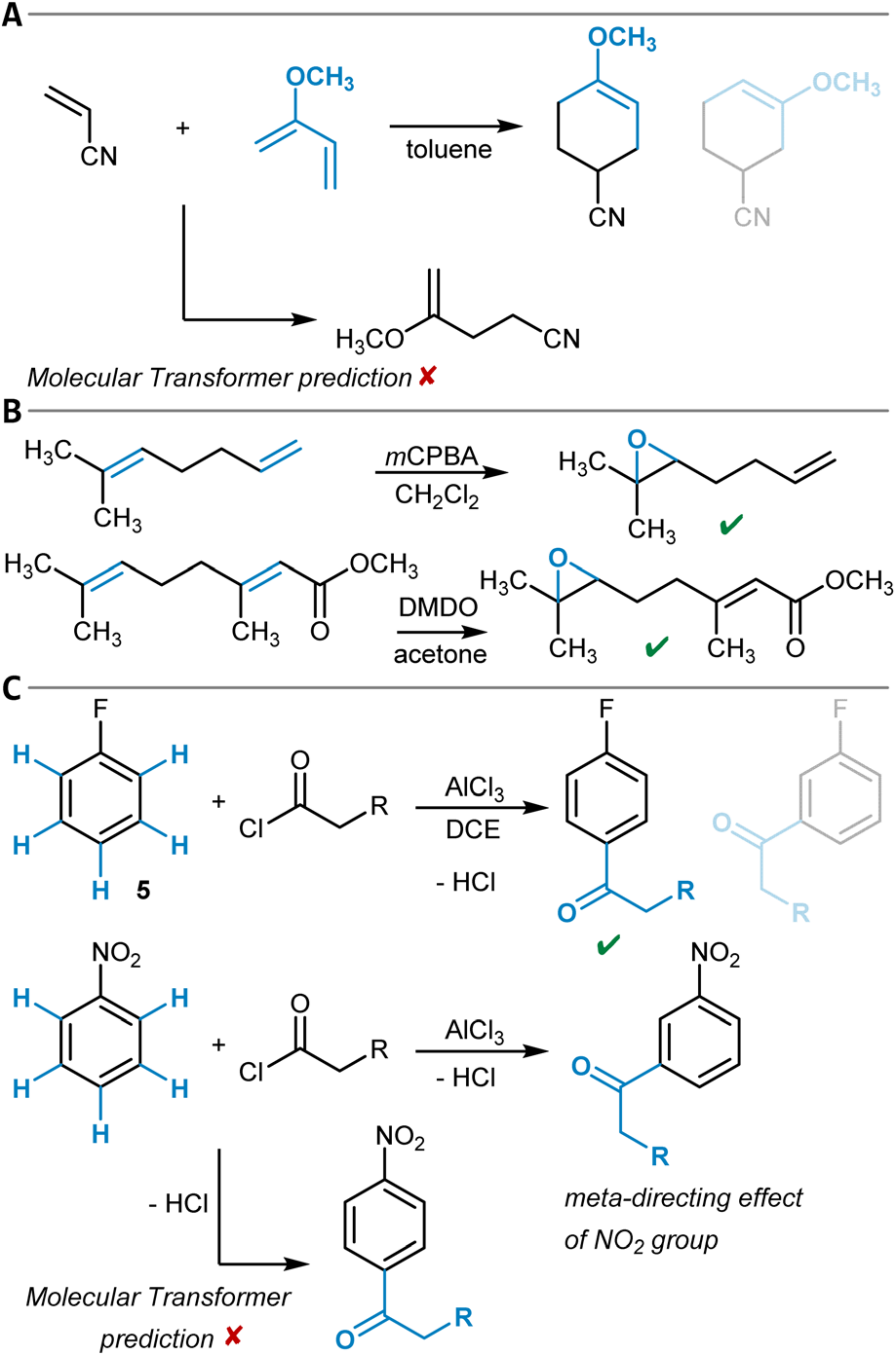
(A) Diels–Alder reaction of acrylonitrile and 2-methoxybuta-1,3-diene with the main reaction product shown first and the alternative possible regioisomer depicted half-transparently second. The Molecular Transformer failed to recognize the Diels–Alder reaction. (B) Two alkene epoxidation reactions that are both predicted correctly by the Molecular Transformer. (C) Friedel–Crafts acylation of fluorobenzene with the main reaction product shown first and an alternative possible isomer depicted half-transparently second, which was correctly predicted by the Molecular Transformer (top). Expected *meta*-directing influence of the nitro group during the Friedel–Crafts acylation of nitrobenzene and the respective Molecular Transformer output that predicts *para*-substitution (bottom).

One example of a general synthesis prediction software that was actually tested for its accuracy in site- and regioselectivity prediction is the Molecular Transformer (Table 1, entry 1).^101,102^ It is a general ML model for the prediction of organic reaction outcomes based on the transformer architecture,^91^ which takes the SMILES string of the reactants and reagents as input and predicts the SMILES string of the product. Applied to a general test set of reactions, an accuracy of 90% was achieved, which naturally includes a variety of different regio- and site-selectivity questions. The developers of the Molecular Transformer also tested their model separately on a test set of 445 electrophilic aromatic bromination reactions for which only one reaction product was reported. A top-1-accuracy of 83% and a top-2-accuracy of 91% was found. In a separate study that aimed for the rationalization of the Molecular Transformer's decision-making,^103^ deficiencies in handling the regioselectivity of Diels–Alder reactions (Fig. 3A) were discovered while the site-selectivity of alkene epoxidation reactions was predicted accurately (Fig. 3B). Interestingly, a *para*-selective Friedel–Crafts acylation of fluorobenzene (5) was predicted correctly, though strong evidence was found that this is due to the high bias in the training dataset toward *para*-substitution (Fig. 3C). These findings show that detailed training dataset and model robustness analyses are highly important for an in-depth assessment of model performance beyond the standard metrics like accuracy – especially for topics like site- and regioselectivity.

From a fundamental physical perspective, reaction selectivity in general, including regio- and site-selectivity, arises from different energy levels associated with the key transition states. The Curtin–Hammett principle relates the difference in Gibbs free energy of two competing transition states to product ratios and is often used to rationalize and predict selectivity.^104^ General-purpose ML models that predict the activation energies of chemical reactions could therefore also be used for selectivity predictions. While two- and three-dimensional GNN architectures have been developed for activation energy prediction of general chemical reactions,^105–108^ there is currently a lack of sufficient datasets to train foundational^109^ activation energy models.^110^

## C(sp^3^)–H functionalization reactions

The direct functionalization of C(sp^3^)–H bonds is possible with several reaction strategies depending on the chemical environment around the position that is desired to be functionalized. Unactivated C–H groups are particularly challenging to address as they are characterized by extremely low acidity (p*K*_a_ ≈ 50, heterolytic C–H cleavage) and high bond dissociation energy (BDE > 400 kJ mol^−1^ (96 kcal mol^−1^), homolytic C–H cleavage).^111^ Concomitantly, even molecules of moderate complexity often possess several chemically similar C(sp^3^)–H groups, making the design of reactions with high site-selectivity inherently challenging. In this section, we start by discussing radical reactions which include the critical abstraction of a hydrogen atom from the substrate. Next, reactions involving insertion into C(sp^3^)–H bonds are presented, followed by acid–base reactions.

### Radical reactions

The site-selectivity factors of radical substitution reactions of alkanes are well-known and commonly rationalized with Hammond's postulate, which explains, for example, the much higher selectivity of bromination compared to chlorination reactions.^112^ In recent years, more sophisticated synthetic approaches based on radical mechanisms have gained increased attention for C(sp^3^)–H functionalization, sometimes with high site-selectivity.^113^

**Fig. 4:**
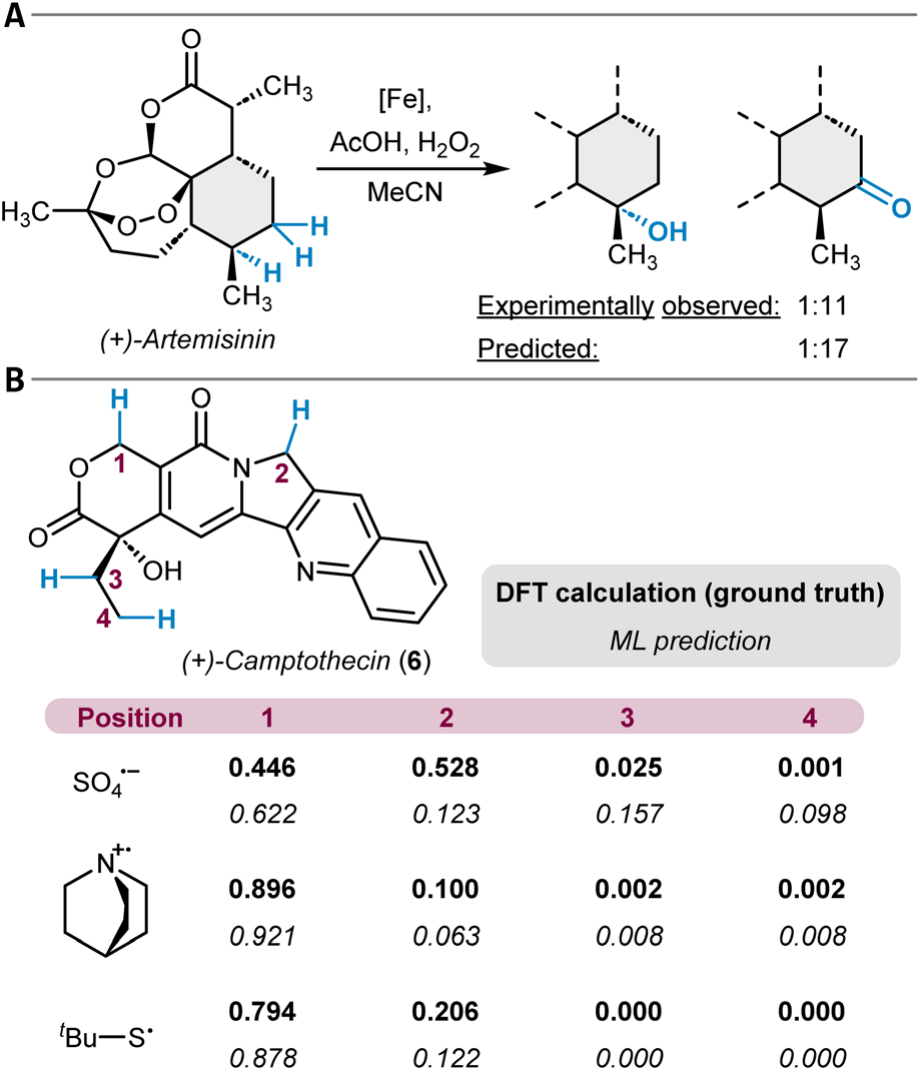
(A) Iron-catalyzed C(sp^3^)–H oxidation of (+)-artemisinin with the experimentally observed site-selectivity and the respective prediction from a linear model. (B) DFT-computed and ML-predicted hydrogen atom abstraction selectivities at (+)-camptothecin with three different abstraction agents.

In the area of hydroxylation, the White group has made significant advances with the development of their Fe(PDP) catalyst.^114^ They derived linear regression models based on atomic partial charges and *A*-values^115^ to fit experimentally determined ΔΔ*G*^‡^ values for two different catalytic systems and applied them to complex natural products (Fig. 4A).^116–118^ Likewise, the labs of Sigman and Movassaghi have developed linear models based on similar features for the prediction of the oxidation site mediated by bis(pyridine)silver(i), which were successfully applied to model systems with more than one potential site for oxidation.^119^ In another study, regression models for high-valent ruthenium-catalyzed oxidation reactions were reported in which the natural σ-bond orbital energy difference of two competing C(sp^3^)–H sites was found to be an important feature.^120^

ML tools beyond multiple linear regression (MLR) for unactivated C(sp^3^)–H functionalization reactions were also developed. Hong and coworkers have built an ML model for the prediction of activation barriers of hydrogen atom transfer reactions (HAT) through photoredox catalysis.^121^ A dataset of 2962 computed DFT barriers for various HAT examples (mainly of C(sp^3^)–H bonds) was used to train models with physical organic chemistry descriptors for local and global properties of both the substrate and the corresponding hydrogen atom abstracting reagent. The selection of features included atomic charges, C–H BDEs, buried volumes, and Wiberg bond indices as well as descriptors for the frontier molecular orbitals of the reactants. The trained AdaBoost model was tested against experimentally determined free energy barriers for a set of 117 examples resulting in a mean absolute error (MAE) of 2.8 kJ mol^−1^ (0.7 kcal mol^−1^). Furthermore, the authors applied their tool to challenging substrates such as (+)-camptothecin (6) with three different radicals (Fig. 4B). The different reagents alter the site-selectivity, and the correct major site of reactivity was predicted for two of the three cases. A closely related study reported on linear and neural network models for the prediction of HAT activation barriers with alkoxy radicals by using similar features as just described. Here, the trained neural network model was able to correctly predict the preferred site of reactivity for a set of six small hydrocarbons.^122,123^ Another example in this context is a study on dehydrogenation reactions of hydrocarbons in which DFT-simulated nuclear magnetic resonance (NMR) chemical shifts were used as features for modeling site-specific reaction rate constants.^124^

Very recently, the groups of Milo and Reisman published on RF models including active learning applied to the site-selectivity prediction of oxidation reactions with dimethyldioxirane and methyl(trifluoromethyl)dioxirane.^125^ A dataset of 185 substrate molecules was used, and the individual C–H positions were described with steric (percent buried volume, pyramidalization), electronic (NMR chemical shifts and C–H BDEs), and structural (neighboring atoms and their hybridization) features. A leave-one-out top-1 accuracy of 80% was reported.

One of the most informative substrate descriptors in the context of radical C(sp^3^)–H functionalization is the BDE of the respective C–H group as weaker bonds tend to be more prone to react. Several statistical tools have been developed for the prediction of BDEs. Different model architectures like RF and related tree-based algorithms,^126,127^ support vector machines,^128^ various neural network architectures,^129–132^ and hybrid approaches between SQM and ML^133^ were employed. Some works focused on certain functional groups or extended existing models to a larger chemical space.^134^ Predicted BDEs from these tools can be used to study C(sp^3^)–H functionalization reactions, possibly in conjunction with additional site-specific descriptors.^135^

One example of the application of these methods is a small case study conducted by Paton and coworkers.^130^ They used their GNN ML model ALFABET to identify the weakest C–H bond in each of 28 small molecule drugs, which were the subject of site of metabolization studies.^136^ They showed that the model is as accurate as DFT-calculated BDEs for identifying positions of oxidative metabolization. Apart from this example, significant research effort has been devoted to site of metabolization predictions, for example of cytochrome P450-related processes.^137–139^

### Reactions of nitrenoids and carbenoids

**Fig. 5:**
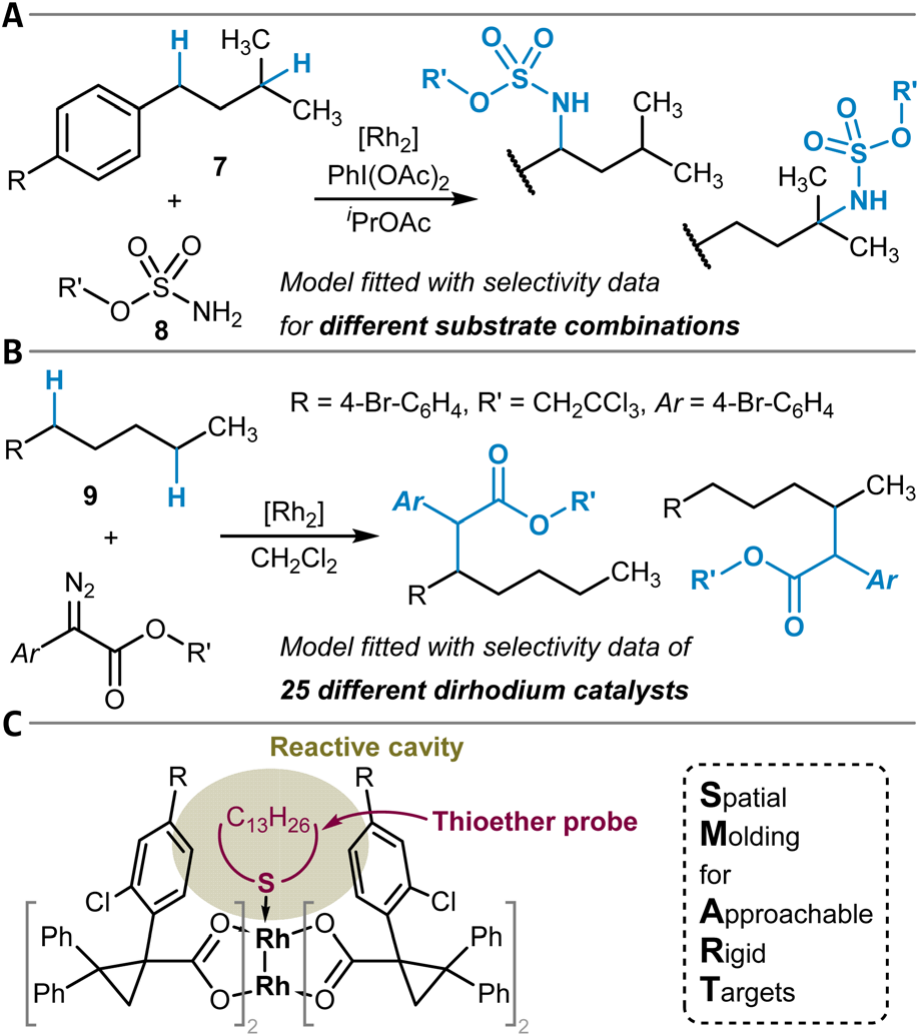
Dirhodium complex-catalyzed formation of (A) C(sp^3^)–N and (B) C(sp^3^)–C bonds by insertion reactions into C(sp^3^)–H bonds. (C) SMART featurization approach to model the spatial accessibility of the reactive cavity of the Rh_2_-tetracarboxylate catalysts through the conformational flexibility of the macrocyclic thioether probe attached to the dirhodium catalytic system.

Another strategy for the direct chemical modification of unactivated C(sp^3^)–H groups is to target them with highly reactive organometallic or closely related species that can insert into the C–H bond. The formation of new C(sp^3^)–N bonds can be mediated by transition metal nitrene complexes, and the site-selectivity of respective reactions was studied computationally in several instances.^140–142^ Furthermore, MLR models can predict the site-selectivity of dirhodium-catalyzed amination reactions of isoamyl benzenes 7 with different sulfamate esters 8 as nitrene precursors, as shown by Du Bois, Sigman, and coworkers (Fig. 5A).^143^ Key features for these models were selected normal mode vibrational frequencies and intensities of the sulfamate esters obtained through DFT calculations.^144^ The model was employed to identify sulfamate esters that preferentially lead to benzylic amination.

Alternatively, carbenoids, also based on dinuclear rhodium complexes, can enable C(sp^3^)–C(sp^3^) bond formation by insertion into C–H bonds.^145^ The research groups of Davies and Sigman have developed several MLR models for this class of reactions (Fig. 5B). In a first study, experimentally determined ΔΔ*G*^‡^ values for site-isomeric reactions were regressed by also making use of quantum chemically calculated vibrational frequencies (intensity of the diazo esters' N=N stretching vibration) in combination with NBO partial charges.^120^ Later, additional models were built for related Rh_2_ catalytic systems based on the newly invented SMART (Spatial Molding for Approachable Rigid Targets) steric descriptors (Fig. 5C).^146^ These are obtained by *in silico* attachment of a flexible macrocyclic thioether probe to the catalyst and subsequent constrained conformational analysis. From the resulting ensemble of conformers, a collection of descriptors is derived, for example, the cavity volume of the cone-shaped catalysts. In contrast to the amination reaction (see above) for which the models were trained with the data of different substrates and the same catalyst, the models here were developed and evaluated using a library of 25 different catalysts for the functionalization of one substrate (1-bromo-4-pentylbenzene (9), Fig. 5B).

For silyl ether substrates, logistic regression models were established for two different Rh-catalysts based on the energy difference between the σ and σ* orbital (derived from natural bond orbital calculations) of the substrate's C–H bond, the respective ^1^H NMR chemical shift, and the relative buried volume around the carbon atom.^147^ The models were trained and tested on sets of 157 and 114 different silyl ethers and test accuracies of more than 95% were reported. The authors also applied one of the models to evaluate a complex steroid substrate with more than 20 candidate sites for functionalization and successfully identified the major reaction product.

In related work, Besora *et al.* derived regression models for silver-mediated aliphatic hydrocarbon functionalization through carbene insertion reactions with three different catalytic systems.^148^ Both, quantum chemically obtained features (*e.g.*, orbital and BDEs) as well as topological descriptors (*e.g.*, degree of substitution or number of carbon atoms in the attached hydrocarbon chain) for a given C–H group allowed to model ΔΔ*G*^‡^ values determined through experiments, with an *R*^2^ score of above 0.93. It was also possible to transfer the methodology, called QDEAN (quantitative descriptor-based alkane nucleophilicity), to similar reactions catalyzed by copper or rhodium.

### Acid–base reactions

**Fig. 6:**
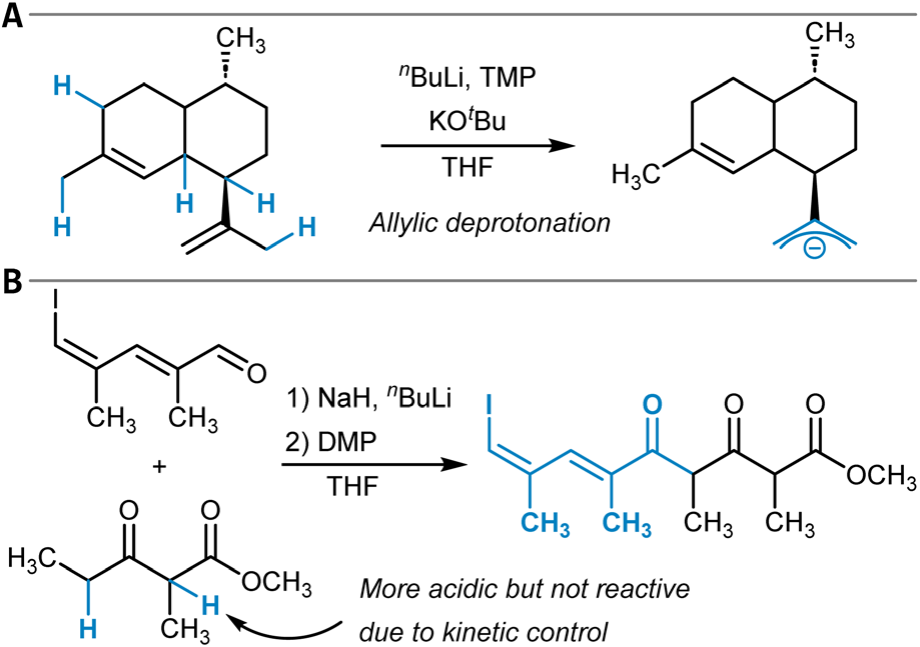
(A) Site-selective deprotonation of an allyl group that determines the selectivity of the following oxidation reaction (*cf.* ref. 149). (B) Aldol reaction followed by oxidation with the Dess–Martin periodinane (DMP). The kinetically controlled reaction product is formed due to deprotonation of the methylene group.

The previous sections discussed the predictive modeling of functionalization reactions of unactivated C(sp^3^)–H groups through radical and insertion mechanisms. However, placing aliphatic C–H groups in close vicinity to carbanion-stabilizing groups renders the respective protons increasingly acidic and consequently enables chemical modifications by acid–base chemistry, for example, through aldol-type transformations. At the same time, the site-selectivity question as introduced at the beginning of this section remains relevant because the site of deprotonation determines the reaction outcome (Fig. 6).^149^ Accurate p*K*_a_ assessments are also important for many research areas beyond C–H functionalization, for instance, to determine the preferred position of protonation in Brønsted acid-catalyzed reactions. Hence, *in silico* p*K*_a_ prediction across various functional groups has been explored extensively in the past and was reviewed by Wu *et al.*^150^ Very recent additions to this area are for example, the QupKake model,^151^ which combines GFN2-xTB calculations with GNNs for p*K*_a_ predictions or Uni-p*K*_a_, which relies on a transformer architecture operating on three-dimensional molecular structures.^152^

Roszak, Beker, and others developed ML models specifically for the prediction of p*K*_a_ values of C–H groups using a dataset of 822 molecules, including 414 experimentally obtained datapoints.^153^ The most accurate model was found to be a graph convolutional neural network supplied with atom features like atomic numbers, hybridizations, electronegativities, or Gasteiger partial charges, achieving an MAE of 2.2 p*K*_a_ units for the test set. The model was applied to a large collection of 12 873 reactions, and the correct site of reactivity was identified in 90.5% of the cases. The final computational tool was also supplemented with several hand-crafted structural analyses which, for example, inform the user on the potential presence of directing groups resulting in the deprotonation of an *a priori* less acidic position or output warnings for sterically encumbered sites.

Borup *et al.* trained LightGBM models for the identification of the most acidic C–H site in organic molecules.^154^ As features, CM5 atomic partial charges calculated at the GFN1-xTB computational level were used, and the resulting model showed an MAE of 1.24 p*K*_a_ units (Table 1, entry 2). The authors used the model to predict selectivities for several reaction types, *e.g.*, for an aldol reaction (Fig. 6B).^155^ They discussed the interplay between thermodynamic and kinetic control in deprotonation reactions of C–H acidic compounds and noted that their model identifies the site of lowest p*K*_a_, which can result in incorrect selectivity predictions for kinetically controlled reactions. In a recent and closely related follow-up study, similar ML models were trained for the prediction of C–H hydricity, that is, the heterolytic bond dissociation Gibbs free energy to give a carbocation and a hydride anion (H^−^).^156^

## Aromatic C(sp^2^)–H functionalization reactions

The diversification of aromatic C–H groups is certainly among the most prevalent chemical transformations across all organic chemistry. Broadly speaking, C_aromatic_–H functionalization reactions may be grouped into polar reactions (which are the electrophilic aromatic substitutions), radical reactions, and C–H activation-mediated transformations. We will here present site-selectivity prediction tools for all three reaction classes, starting with the polar reactions. Of note, nucleophilic substitution reactions can in certain cases also be used to modify C_aromatic_–H groups.^157^ A respective selectivity model including the vicarious nucleophilic substitution reaction is mentioned in the section on nucleophilic aromatic substitutions (see below).^158^ In general, nucleophilic C–H functionalizations are less recognized, and their site but also chemoselectivity could be the objective of future research.

### Electrophilic aromatic substitution reactions

**Fig. 7:**
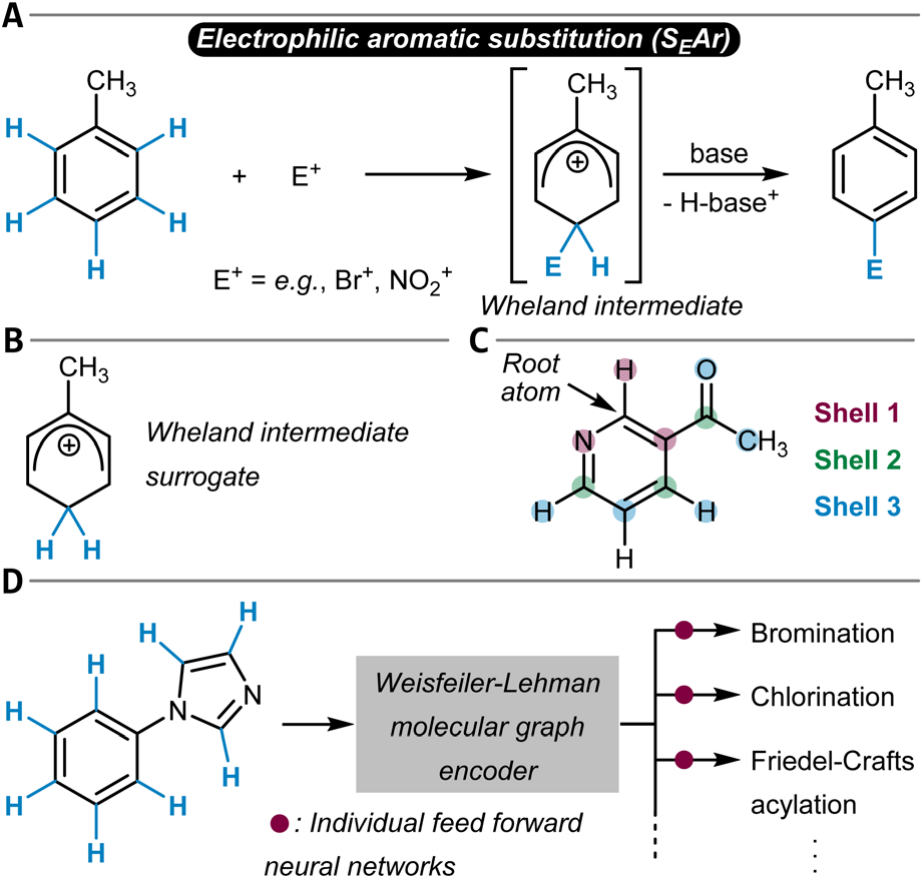
(A) Schematic reaction mechanism of an electrophilic aromatic substitution reaction (S_E_Ar). (B) The relative stability of protonated aryl substrates can be used as a surrogate of the real Wheland intermediate for site-selectivity predictions. (C) Shell-wise local featurization of atomic positions. (D) Multitask site-selectivity prediction in which the Weisfeiler-Lehman encoder learns molecular embeddings which are passed to separate feed-forward neural networks for reaction-specific site-selectivity prediction. During training, the entire model (graph encoder + readout networks) is optimized simultaneously.

Electrophilic aromatic substitution reactions (S_E_Ar) have been extensively studied with the first site-selectivity prediction guidelines dating back more than 120 years from today.^159,160^ With the advent of QM and its application to chemical reactivity, the S_E_Ar reaction was investigated from a combined experimental and theoretical perspective.^161,162^ Although alternative and more sophisticated reaction mechanisms were discussed,^163^ the rather simple two-step mechanism as shown in Fig. 7A including the Wheland intermediate^164,165^ is the main model used to understand and predict S_E_Ar reactions and their site-selectivity.

Many local descriptors obtained through QM calculations^166,167^ were developed for, or applied to S_E_Ar reactions – aiming for the prediction of their site-selectivity. Examples are the well-known condensed Fukui indices^168–172^ and thereof derived parameters,^173–176^ C–H bond strengths,^177^ (group) electronegativities,^171,178^ electrophile affinities,^179^ electrostatic potentials,^179^ activation hardnesses,^180^ atomic partial charges,^172,181–183^ quantities derived from reactive hybrid orbitals^184^ and from the quantum theory of atoms in molecules (QTAIM),^185,186^ or the average local ionization energy.^187^

Liljenberg *et al.* compared relative Wheland intermediate stabilities with average local ionization energy data for the quantitative prediction of product distributions of electrophilic halogenations, nitrations, and Friedel–Crafts acylations.^188^ Halogenation reactions were mostly predicted successfully by both approaches, while nitrations and acylations were found to be more problematic. The authors highlighted the importance of the inclusion of explicit solvent molecules or reaction conditions for accurate reaction modeling. A similar approach was later also pursued for the nucleophilic aromatic substitution reaction (see below).^189,190^

The publications referenced in the preceding paragraph demonstrate how physics-based modeling assists in predicting and understanding the site-selectivity of S_E_Ar reactions. However, practical (ML) tools for site-selectivity building on simulations were only developed in recent years. For early approaches of practical use, it was common to rely on empirically derived decision rules. The synthesis planning software CAMEO^98^ for example, which was developed in the 1980s, had an S_E_Ar module that included an MLR model along with several other decision rules for selectivity prediction.^191^

Much later, the RegioSQM tools^84,192^ (Table 1, entry 3) were introduced, which predict the site-selectivity of S_E_Ar bromination reactions with fast SQM simulations by calculating the proton affinities of the individual C_aromatic_–H positions at the PM3 or GFN1-xTB computational level (Fig. 7B).^193^ The lowest-energy structure of the protonated substrate (relative proton affinity) identifies the reactive position as a surrogate for the Wheland intermediate.^194^ The model was applied with an accuracy of 93% to 535 reactions extracted from the literature. However, also the explicit calculation of halenium ion affinities (*e.g.*, Cl^+^, Br^+^) was used for regio- and site-selectivity and also chemoselectivity predictions.^195^

RegioSQM does not include an ML component and instead makes deterministic site-selectivity predictions from the calculated proton affinities. Interestingly though, Elrod, Maggiora, and Trenary provided already in 1990 an early proof-of-concept study for the application of ML for site-selectivity prediction of S_E_Ar reactions to introduce a second substituent to monosubstituted benzene rings.^196^ They trained small neural networks using an atom connectivity table of the first substituent or atomic partial charges obtained from SQM calculations of the six benzene ring atoms as model inputs to predict the relative isolated amount of combined *ortho*/*para*- and *meta*-substitution product. A similar contribution was made for electrophilic aromatic nitration reactions.^197^

Further building on the philosophy of combining QM data with ML, Tomberg *et al.* developed models for the classification of aromatic C–H groups into “reactive” and “unreactive” in S_E_Ar reactions – also going beyond bromination (Table 1, entry 4).^198^ The features for the individual C_aromatic_–H groups were atomic partial charges, bond orders, condensed Fukui coefficients, solvent-accessible surface areas, and proton affinities, computed at the DFT level for a dataset of 694 molecules. An RF classifier with an accuracy of 93% (per C–H group) was found to be the most accurate for the classification task. Although trained mainly on bromination reactions, it was shown that the model generalized well to chlorination (94% accuracy). Iodination was less accurately predicted (66% accuracy), most likely due to the markedly different reaction mechanism.

While the above-mentioned descriptors, which are rooted to a respective carbon atom, are naturally the result of that atom's chemical environment, explicit information on neighboring atoms or bonds is not included. To achieve that, Ree and others applied an atomic partial charge shell featurization technique to describe the individual sites of a molecule (Fig. 7C).^199^ The atomic charges for 21 896 bromination reaction substrates were obtained from GFN1-xTB calculations and were arranged in five concentric shells around the aromatic carbon atom of interest.^200^ Within the individual shells, the substituents were ordered following the Cahn–Ingold–Prelog rules. The resulting tool was called RegioML (Table 1, entry 5), is based on the LightGBM algorithm, and achieves accuracies of above 90%.

Another approach to consider information on neighboring atoms during the prediction of site-selectivity is to apply GNNs, as the message-passing steps distribute information across the molecular graph. Struble, Coley, and Jensen demonstrated this for a family of S_E_Ar reactions (bromination, chlorination, nitration, and sulfonylation) and also more generally for all transformations of a C_aromatic_–H group into a C_aromatic_–R group.^201^ They categorized their dataset of 58 000 individual reactions into 127 unique classes and used it to train a Weisfeiler-Lehman GNN (WLN)^202^ encoder coupled to feed-forward neural networks for predicting the probability of a given C–H group to be the preferred site of reactivity (Fig. 7D and Table 1, entry 6). Atom features were for example atomic number, Gasteiger charge, atomic contributions to Crippen log *P*, or accessible surface area, and bond descriptors were bond order and ring status. All read-out networks (one for each reaction class) were trained together with the shared WLN encoder weights, which was found to increase the prediction accuracy of the model (84% top-1 test set prediction accuracy for the multitask model compared to 81% for the single task model).

Going beyond DFT as a source of features, NMR chemical shifts are directly obtainable from experiments (in addition to the possibility of quantum chemical derivation). At the same time, they are site-specific descriptors that are highly diagnostic of the local electronic structure. Kruszyk and others have utilized the NMR chemical shift predictions of the ChemDraw software to build an S_E_Ar model for heteroaromatic systems (bromination reactions) by simply identifying the lowest ^13^C or ^1^H shift value.^203^ Though a few ring types were not handled well by the model, this method allows a quick and straightforward assessment of heteroaromatic substrates in bromination reactions and demonstrates the prognostic capabilities of NMR chemical shifts in the context of site-selectivity prediction.^204,205^ In fact, several following research efforts have picked up this feature to build more advanced models.

Paton and coworkers have deployed their ^1^H and ^13^C NMR chemical shift ML model CASCADE to produce features for an RF classifier for the prediction of S_E_Ar reactions of 75 small organic molecules.^206^ The combination of the predicted chemical shifts of the C_aromatic_–H group under consideration with data from RegioSQM^84,192^ (see above) gave a model with 91% accuracy.

The Green and Jensen groups further developed the approach of using ML regressors of chemical descriptors as input generators for site-selectivity models.^62^ For that, they initially trained a multitask GNN regression model for molecular site descriptors like atomic partial charges, condensed Fukui indices, or partial bond orders with ground truth data from DFT.^65,207^ The predicted descriptors from this model were then used as features for the training of different site-selectivity models, denoted ml-QM-GNN (Table 1, entry 7). Importantly, features for the substrate and the key reagent (*e.g.*, *N*-bromosuccinimide in a bromination reaction) were included in the model architecture. To train and test their tool, the extracted reaction data was categorized into three individual classes: C_aromatic_–H functionalization (mainly S_E_Ar), C_aromatic_–X functionalization (mainly S_N_Ar, see below), and a more general group of reactions. Accuracies in predicting the correct major isomer of 90%, 97%, and 97% were achieved, respectively.

In a following study, it was shown that it is important for the ml-QM-GNN model to be supplied with physics-related features that cover both the electrostatic and orbital interactions of the reactants.^63^ It was also highlighted that especially in very low data regimes, detailed mechanistic considerations such as protonation of the substrate molecule due to strongly acidic conditions or a change in reaction mechanism are of great importance. When only little reaction data (≈200 datapoints) is available, ml-QM-GNN models trained with DFT features cannot learn the implications associated with a certain functional group (*e.g.*, protonation of aniline derivatives). These modifications result in a significant change in the electronic structure of the molecule, which is not covered by the parent feature vector, *e.g.*, of the neutral amine. The application of larger datasets (given their availability, ≈2000 datapoints) can counteract this source of error as the model can implicitly learn the influence of different functional groups.

In a more fundamental approach, deep neural network architectures can be used to directly model the potential energy surface of molecules, one example being the family of AIMNet models.^208^ Zubatyuk *et al.* expanded the capabilities of AIMNet to handle arbitrary combinations of molecular charge and spin multiplicity (AIMNet-NSE).^209^ This not only gives access to the respective molecular electronic energies but also to atomic partial charges from which C-DFT descriptors like Fukui coefficients can be derived. They used the features from AIMNet to retrain Tomberg's RF model^198^ (see above) and reported a validation set accuracy of 90%, on par with the original model.

### Radical reactions

**Fig. 8:**
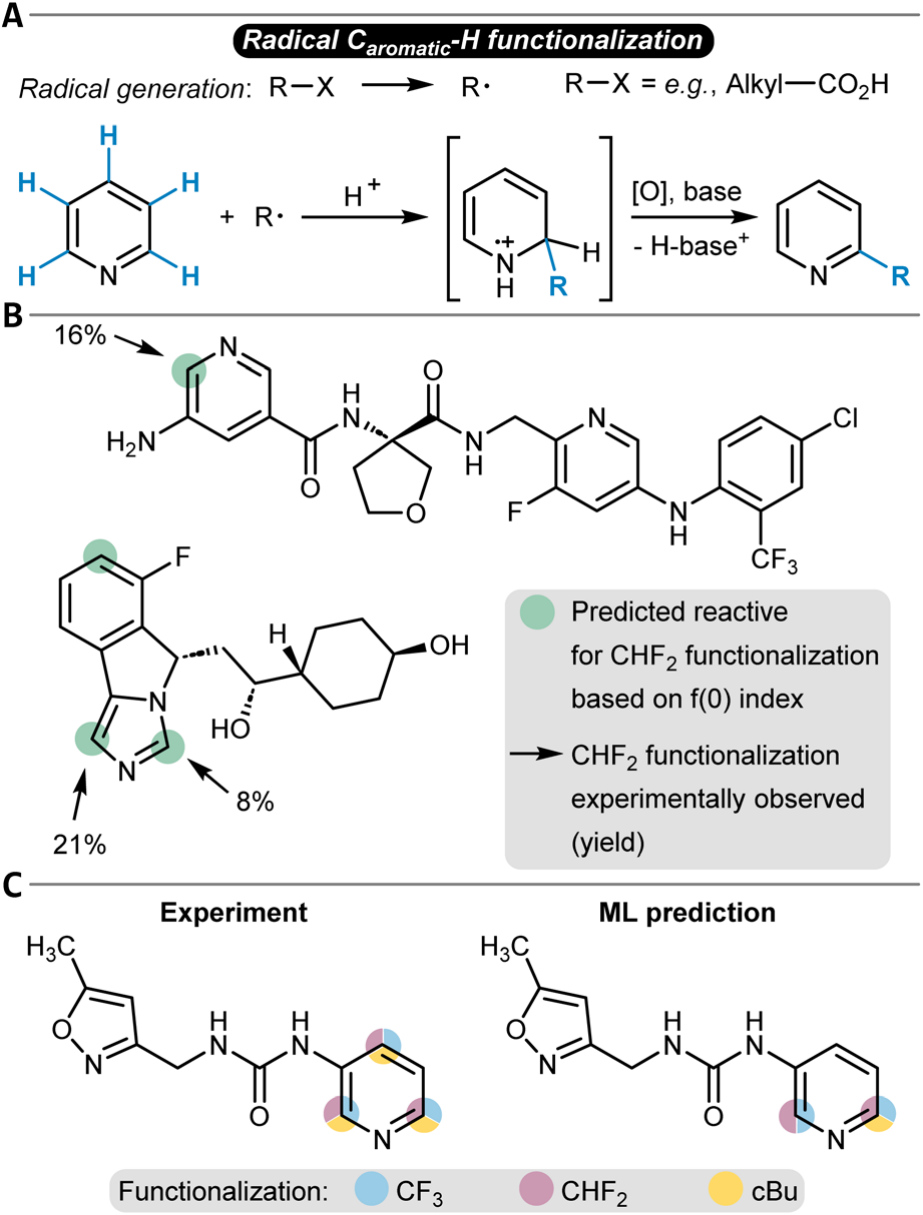
(A) Schematic reaction mechanism of a radical C_aromatic_–H substitution reaction. Predictions of respective functionalization reactions with (B) the Fukui index for radical attack, *f*(0), and (C) with a GNN ML model and comparisons to experimental observations.

Several approaches have been used to predict the site-selectivity of aryl C–H functionalization reactions that follow a radical reaction mechanism. This class of reactions offers a valuable alternative to S_E_Ar reactions as it can target a different substrate scope and introduces different functional groups. Concomitantly, radical functionalization reactions can proceed with high selectivity.^210,211^ The reaction starts with the formation of a (carbon-centered) radical, which undergoes addition to the substrate, forming a radical adduct (Fig. 8A). The adduct then undergoes an oxidative re-aromatization step to restore aromaticity. Mild radical reactions have been developed involving light-mediated radical generation.^212,213^

Similar to other reaction classes discussed herein (*e.g.*, S_E_Ar), a popular method to predict selectivity in radical reactions has been the utilization of descriptors obtained from quantum chemistry. Atomic charges, in particular, represent a powerful descriptor to gain insight into the reactivity of each site of the substrate, as for example shown for bromination reactions involving an acridinium-based photocatalyst.^214^ Fukui indices were used in a similar fashion to rationalize the site-selectivity of several types of derivatizations, such as amination reactions, and were also shown to align with handcrafted site-selectivity rules.^215–218^ Importantly, Fukui indices were also able to predict the correct site-selectivity for late-stage functionalizations of heteroarenes with commercially available Baran Diversinates™ (Fig. 8B).^219^

The availability of a relatively large quantity of data and the relevance of this class of reactions in the pharmaceutical industry made radical C_aromatic_–H functionalization a fertile ground for the generation of several data-driven methods for site-selectivity predictions. In particular, as for other reaction classes, two main strategies have been used within data-driven predictions. The first focuses on the prediction of DFT activation barriers, which are time-consuming to compute, and the second on the pure data-driven prediction of experimentally observed site-selectivities.

The work of Li and others belongs to the first class. They studied the site-selectivity of radical additions to heteroarenes to afford C–H functionalizations with an ML approach that predicts DFT Gibbs free activation energies (Table 1, entry 8).^220^ Comparing the energy barrier of potential radical addition sites gives a clear indication of the site-selectivity because the radical addition step is selectivity-determining.^221^ A dataset of 3406 radical C–H functionalization reactions was used to train several models, involving featurization techniques like topological fingerprints, ACSF, or SOAP as well as descriptors from physical organic chemistry (frontier molecular orbital energies, atomic charges, buried volumes, NICS values, and Wiberg bond indices). The models constructed with physical organic chemistry descriptors were found to perform well while having a smaller feature space. An RF model trained with these features was able to correctly predict the energy difference in DFT barriers with an MAE of 2.1 kJ mol^−1^ (0.5 kcal mol^−1^) ultimately resulting in a prediction accuracy for site-selectivity of 94%. Additional testing on an external dataset revealed a lower accuracy for a chemical space beyond the training set, and the authors suggested that this could be counteracted with an active learning strategy in future work.

More strongly data-driven approaches were also developed, in particular for the prediction of radical late-stage functionalization reactions. The Lee group implemented a GNN model to predict the probability for functionalization for each atom in Minisci-type late-stage and P450-based functionalizations as well as in a small number of photoredox and electrochemical alkylation reactions (Table 1, entry 9).^222^ The model had a GNN architecture and was trained with basic atomic and bond information such as atomic symbol and hybridization, explicit hydrogen count, or bond type. The training data was sourced from internal Pfizer datasets and contained 2600 reactions. While RF baseline classifiers already showed notable accuracies (up to 94%), the authors turned to transfer learning to improve the models even further. ^13^C NMR chemical shifts were selected as the pre-training target, and the GNN was trained with a dataset of around 27 000 carbon NMR chemical shift values. Subsequently, this pre-trained model was fine-tuned to learn the site-selectivity of the radical functionalization reactions, which then was possible with an accuracy of 96% (94% without fine-tuning, Fig. 8C shows a prediction example in comparison to the experimental observations). The overall model used one-hot encoding to account for different reagents, solvents, or further additives. Interestingly, the addition of physics-based features like Fukui indices as node attributes for the GNN did not improve the accuracy, and the simpler atom and bond descriptors were found sufficient.

GNNs were also trained to predict the reaction feasibility of Minisci-type reactions, and the obtained models were applied to identify promising molecules for experimental testing.^223^ The subsequently gathered experimental results agreed in most cases with the expected reaction outcomes based on known site-selectivity rules;^216^ however, a few deviations were found for more complicated substrates. Therefore, future research efforts could target the combination of site-selectivity and reaction feasibility prediction to obtain more powerful radical C_aromatic_–H functionalization models.

### C–H activation reactions

**Fig. 9:**
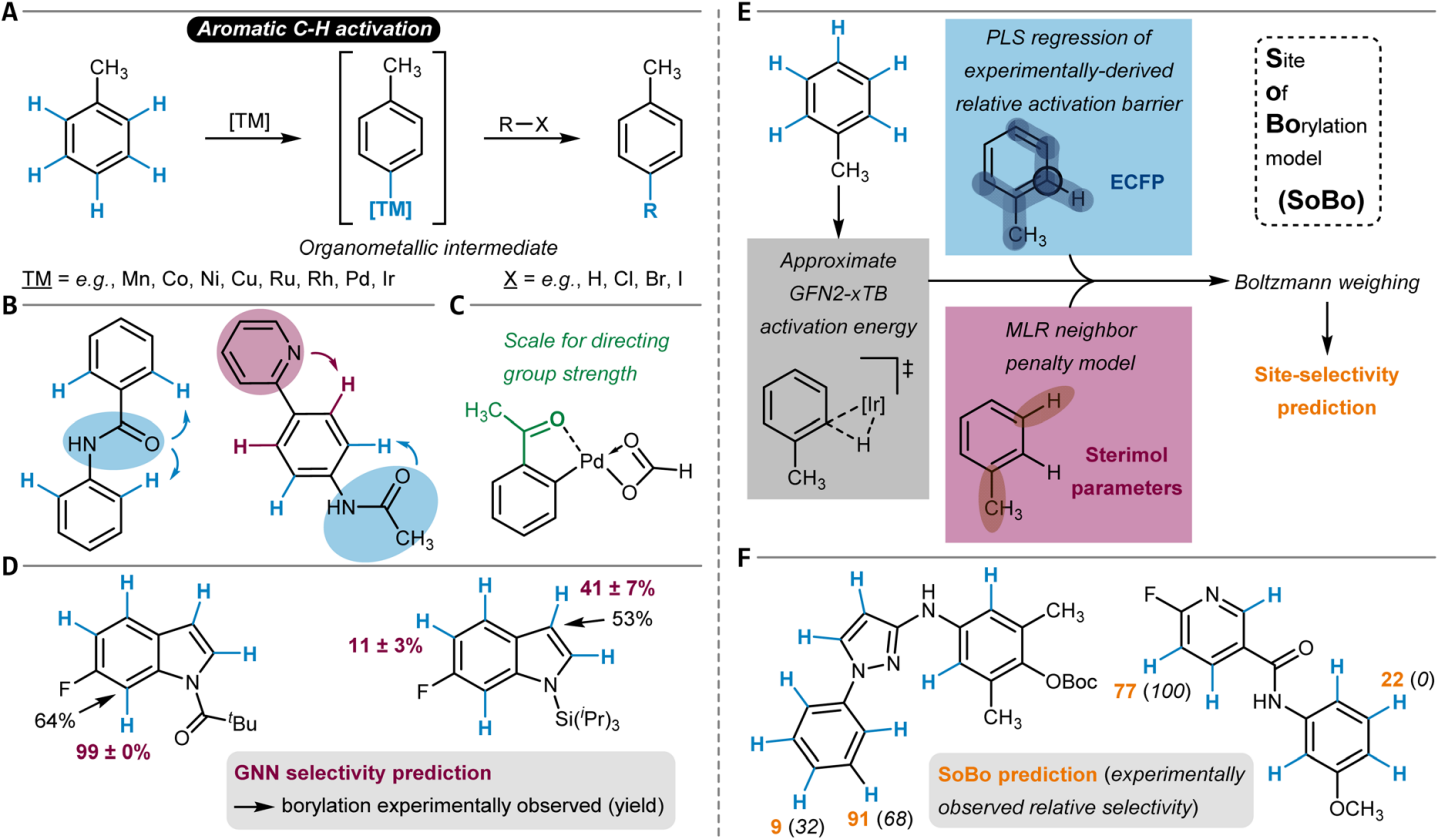
(A) Schematic reaction mechanism of a C–H activation-mediated C_aromatic_–H substitution reaction. (B) A single directing group favoring two different sites (left) and two different directing groups favoring two different sites (right) during C–H activation. (C) Example of the palladacycle intermediate used by Tomberg *et al.* to computationally construct a scale for directing group strength. The example directing group is highlighted in green. (D) Borylation site-selectivity predictions made by a three-dimensional GNN model and comparison to the experimental observations. The predicted percentages and respective standard deviations were obtained by applying the model to ten different conformers of the substrate molecule. (E) Schematic representation of the Site of Borylation (SoBo) model architecture and (F) two of its prediction examples in comparison to the experimental observations.

In comparison to the previous two sections on S_E_Ar reactions and transformations involving radical species, C–H activation – and in this context, C_aromatic_–H activation – goes through an organometallic intermediate, that is a species with a carbon–metal bond (Fig. 9A).^224,225^ This intermediate reacts further to give the reaction product. Transition metal catalysis has proven especially powerful for C–H activation of (hetero)arenes, and depending on the applied catalytic systems, different reaction mechanisms are operative.^226^ Examples are concerted metalation–deprotonation (CMD) or electrophilic aromatic substitution (see above), *e.g.*, within palladium catalysis, or oxidative addition for instance found for iridium-based catalytic systems.

The site-selectivity of C_aromatic_–H activation reactions has been the subject of many quantum chemical studies,^227,228^ and was also discussed in a review article by Davies, Macgregor, and McMullin.^229^ In particular, DFT calculations have been employed to investigate how fluorine substituents on aryl rings affect C–H bond dissociation enthalpies and therefore the site-selectivity of oxidative addition reactions with a broad variety of transition metal complexes, for example, based on nickel, zirconium, rhodium, or iridium.^230^ Pabst and Chirik also considered other reactivity factors such as chelation assistance or steric accessibility and provided an overview of the selectivity-determining factors of C(sp^2^)–H oxidative addition reactions.^231^ For palladium-catalyzed transformations following the CMD mechanism, the deformation energy of the substrate molecule toward the CMD transition structure was found to correctly predict site-selectivity for some substrate classes.^232^ In this context, BDEs calculated from transition structures were also discussed as a selectivity indicator.^233^

To facilitate the computational investigation of palladium-catalyzed C–H activation reactions, Cao *et al.* developed an automated DFT workflow to predict the site-selectivity from the relative Gibbs free energy of key reaction intermediates (Table 1, entry 10).^234^ The procedure includes the differentiation between two possible reaction mechanisms (S_E_Ar and proton abstraction), which allowed its successful deployment to a number of known reactions. Similar work was done for lithiation reactions of C_aromatic_–H bonds.^235^ Palladium-catalyzed C_aromatic_–H activation can also be deployed in an electrocatalytic setup, *e.g.*, for olefination reactions of (hetero)arenes.^236^ An Extra Trees ML model was trained to predict its site-selectivity from descriptors like atomic partial charges, Fukui indices, or BDEs computed for the (hetero)arene substrate (Table 1, entry 11). Additionally, the redox potential of the substrate was considered as a global feature. The model was applied to six separate test molecules and identified the correct main reaction product in all cases.

Many C_aromatic_–H activation reactions benefit from the directing influences of preexisting functional groups. This often simplifies the site-selectivity question and allows for reliable predictions of reaction outcomes. Direction to the *ortho*-position^237^ is most common but depending on the exact nature of the directing group also *meta*^238^- and *para*^239^-C–H activation can be facilitated. The situation gets more complicated either if a given functional group can direct the reaction to two different sites or if a substrate has more than one directing group, each priming different positions for functionalization (Fig. 9B). To tackle such challenges, predictive tools based on DFT calculations have been developed.

Tomberg *et al.* compiled a scale of relative *ortho*-directing strength of 133 different directing groups in palladium-catalyzed reactions (following the CMD mechanism).^240^ This was done by comparing the relative stabilities of the palladacycle intermediates obtained after C_aromatic_–H activation (Fig. 9C). The reactivity metric proved successful in predicting the site-selectivity of 146 out of 150 tested reactions from the literature. In a very recent study, Jensen and coworkers incorporated Tomberg's scale into an automated workflow through SMARTS pattern matching.^241^ Furthermore, they implemented an SQM (and optionally DFT) pipeline to predict the site-selectivity of directed CMD reactions (Table 1, entry 12), which is similar to the one for C–H deprotonation,^154^ electrophilic aromatic substitution^84,192^ (see above), or the Mizoroki–Heck reaction^242^ (see below). An accuracy of 78% on Tomberg's dataset was achieved.

A substituent other than hydrogen in *ortho*-position to the directing group can drastically influence that functional group's directing ability due to steric clashes during C_aromatic_–H activation (*ortho* effect). Tóth *et al.* developed a tool to model such effects based on a structural (dihedral angle, *φ*, along the directing group-arene single bond) and thermodynamic parameter (electronic energy required to set *φ* to 0°).^243^ This allowed them to correctly predict the site-selectivity of palladation reactions of differently substituted *N*-phenylbenzamides.

Besides many palladium-catalyzed transformations, the iridium-catalyzed direct borylation through C_aromatic_–H activation is one of the most important reactions among all C–H activation procedures. This is because it can be used to synthesize starting materials for Suzuki–Miyaura cross-coupling reactions to install new C–C bonds.^244^ Its site-selectivity is strongly governed by steric influences, but also electronic factors such as the acidity of the C–H group can come into play for positions with comparable spatial accessibility.^245^ Furthermore, ligand influences can be of relevance.^246,247^ A collection of empirically derived selectivity rules was compiled, which for some cases were supported with QM simulations.^21,248^

In general, the oxidative addition step of the C_aromatic_–H bond to the iridium catalyst is selectivity-determining. The distortion/interaction model was applied to this elementary step in a similar fashion as it was done for Pd-catalyzed reactions^232^ (see above), and it was found that mainly the interaction energy between the catalyst and substrate influences site-selectivity.^249^ Very recently, three contributions were made targeting a stronger data-driven approach to predicting site-selectivity of C_aromatic_–H borylation reactions. These encompass a model combining classical ML with mechanistic modeling through quantum chemistry, a GNN-based tool, and a fine-tuned language model.

Based on a dataset of 101 iridium-catalyzed borylation reactions including quantitative information on isomer distributions, Caldeweyher, Elkin, and others have developed the site of borylation (SoBo) model which calculates the Boltzmann weight for the transition state of each possible C_aromatic_–H borylation product (Table 1, entry 13).^250^ To achieve that, it refines the approximate oxidative addition transition state energy computed at the SQM level with the output of two ML models (Fig. 9E). The first one is a partial least squares (PLS) regressor trained to predict experimentally-derived relative activation barriers from atom-rooted connectivity fingerprints. The second one is an MLR model that accounts for substituents in *ortho*-position to the currently treated site through Sterimol parameters. The relative influence of the PLS and MLR model is determined through a mixing function. Overall, an accuracy in predicting borylation site-selectivity of 97% was reported. The SoBo tool was applied to six pharmaceutically relevant polyheteroarenes, and in all cases, the correct major site of borylation was identified (Fig. 9F).

The reactivity models of Nippa *et al.* were also mainly trained with iridium-catalyzed reactions but also covered further borylation procedures.^251^ Besides reaction feasibility and yield as target quantities, they implemented different GNNs for site-selectivity prediction (Table 1, entry 14). Training on three-dimensional molecular graphs (steric information taken into account) was shown to be more accurate during model testing compared to the two-dimensional case. At the same time, the inclusion of DFT-calculated atomic partial charges did not significantly improve the prediction accuracy when added to the basic atom features like atom, ring, or hybridization type. The study used a carefully curated literature dataset containing 1301 reactions and an additional dataset with 956 reactions obtained through HTE. Correct site-selectivity prediction results for the literature test dataset were obtained in 90% of the cases. Among a variety of use cases, the authors demonstrated the applicability of their model in examining electronic and steric substituent effects, using the indole scaffold as an example, and predicted the corresponding borylation selectivities correctly (Fig. 9D).^252,253^ Of note, the GNN models were implemented such that also C(sp^3^)–H borylation can be treated within the same model, although the currently available data has a strong bias toward C(sp^2^)–H functionalization.^251^ Future research efforts could work against this imbalance, which will plausibly allow for the construction of more broadly applicable site- and chemoselectivity borylation models.

Kotlyarov *et al.* explored the applicability of the transformer language model T5Chem^254^ to predict C_aromatic_–H borylation reactions by fine-tuning it with a dataset of 1041 iridium-catalyzed aromatic borylation reactions sourced from Reaxys (Table 1, entry 15).^255^ The best model was a classifier predicting each aromatic C–H bond as either reactive or unreactive. This was possible with an accuracy of 95% per bond, which translated to 84% molecule-level accuracy (all bonds within a molecule predicted correctly). The authors also compared their model to the SoBo^250^ and GNN^251^ borylation tool (see above). For three out of the six SoBo test set molecules, the fine-tuned T5Chem classifier labeled all C_aromatic_–H bonds correctly and identified the experimentally observed reaction product. However, the T5Chem model can also classify all bonds as unreactive (as it did erroneously in two of the six cases) allowing for reaction feasibility predictions that are not possible with SoBo as it always predicts at least one site as reactive. When the initial T5Chem model was fine-tuned with the dataset compiled by Nippa and others,^251^ a 94% borylation site-selectivity prediction accuracy was observed, which is higher compared to the 90% obtained with the GNN model.

## C(sp^3^)–X functionalization reactions

The previous two sections of this paper dealt with functionalization reactions directly applied to C–H bonds which are inherently much more prevalent than C–X bonds in most organic molecules. This makes site-selectivity predictions more challenging for C–H bonds. The situation gets simplified when it comes to C–X groups, in which the leaving group X primes the respective position in the molecule for chemical modification. Yet, the X leaving groups must be preinstalled, which can result in additional synthetic effort. Site-selectivity issues only arise when multiple leaving groups are present in the substrate or when the second reaction partner has more than one reactive position. The computational tools that have been developed so far for the prediction of the site-selectivity of C–X functionalizations are discussed now for C(sp^3^)–X and thereafter for aromatic C(sp^2^)–X.

### Bimolecular nucleophilic substitution reactions

A typical reaction class to modify C(sp^3^)–X groups is nucleophilic substitution, in particular bimolecular nucleophilic substitution reactions (S_N_2) in which the leaving group X gets replaced by an incoming nucleophile in a single elementary step.^112^ As indicated above, questions on site-selectivity are generally less common in S_N_2 reactions due to the low prevalence of multiple competing C–X sites in the same molecule. Nonetheless, they are conceivable due to either multiple identical leaving groups within the electrophile or multiple nucleophilic positions within the nucleophile. Broadly applicable tools for either case have so far not been developed but could be targeted in the future.

Besides purely quantum-mechanical computational studies on small model systems,^256–258^ several ML tools for the prediction of S_N_2 reaction rate constants or activation barriers have been reported, for example, with Hammett constants as features or support vector machines as learning algorithm.^259–269^ Their output can in principle be used to predict site-selectivity, given that the model architecture can be (reliably) applied to the system of interest.^270^ However, many studies focused on rather small model systems, which is problematic for the application to larger molecules.

**Fig. 10:**
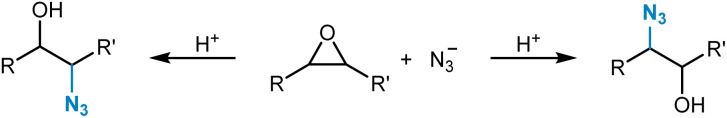
Ring-opening reaction of oxiranes with azide as the nucleophile with the possibility of the formation of two different regioisomers.

One example of a dedicated regioselectivity study for an S_N_2 reaction was provided by Borghini *et al.*^271^ They trained regression models for the prediction of relative selectivity in nucleophilic oxirane ring opening reactions with the azide anion as the nucleophile (Fig. 10). A dataset of 68 reactions was compiled, and for each substrate, one electronic (based on electronegativity) and one steric (based on atomic weight) descriptor was calculated following a concentric atom-shell approach (see Fig. 7C) around the two carbon atoms of the oxirane substrate. A *k*-nearest neighbor model performed best in predicting the relative amount of the experimentally observed reaction product for the test set (*R*^2^ = 0.765).

## Aromatic C(sp^2^)–X functionalization reactions

**Fig. 11:**
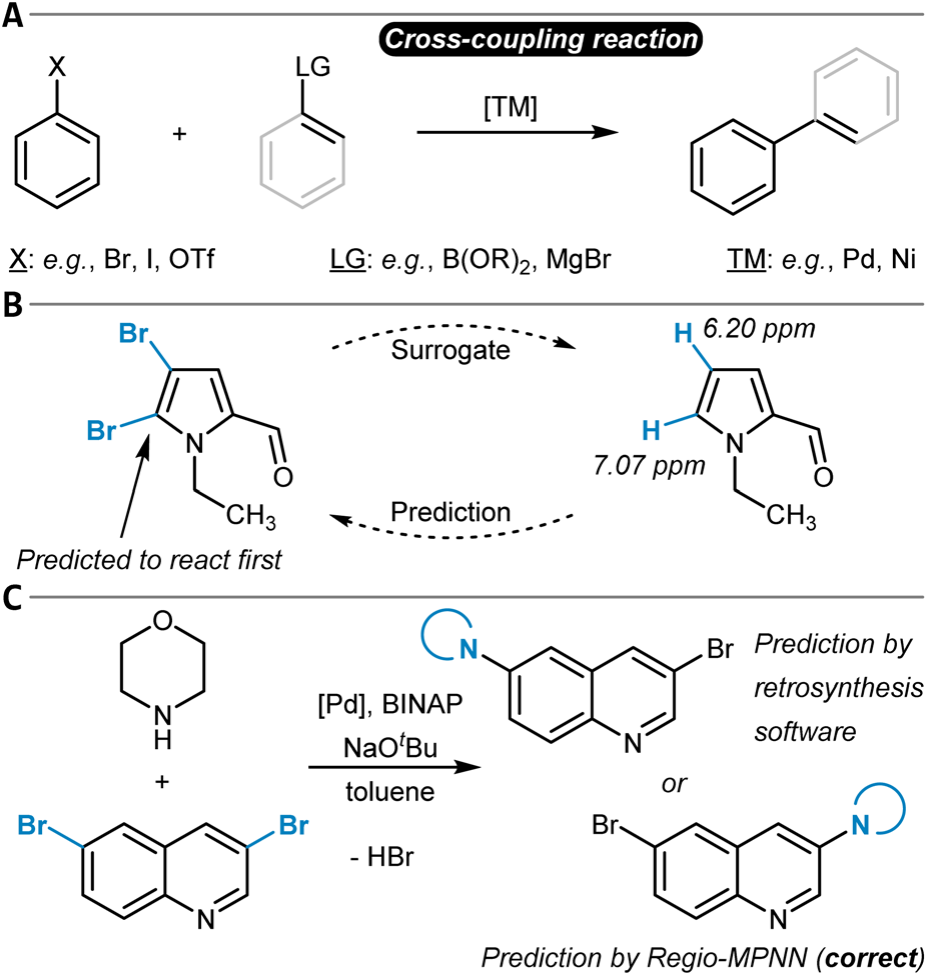
(A) General reaction scheme of a cross-coupling reaction between an aryl halide and an arene or alkene with an appropriate leaving group (LG) catalyzed by a transition metal complex. (B) Handy and Zhang's ^1^H NMR chemical shift model for site-selectivity prediction of cross-coupling reactions. The larger ^1^H NMR chemical shift in the surrogate molecule indicates the reactive position. (C) Application of the Regio-MPNN tool to a Buchwald–Hartwig coupling and comparison to the erroneous prediction of a retrosynthesis planning software.

**Fig. 12:**
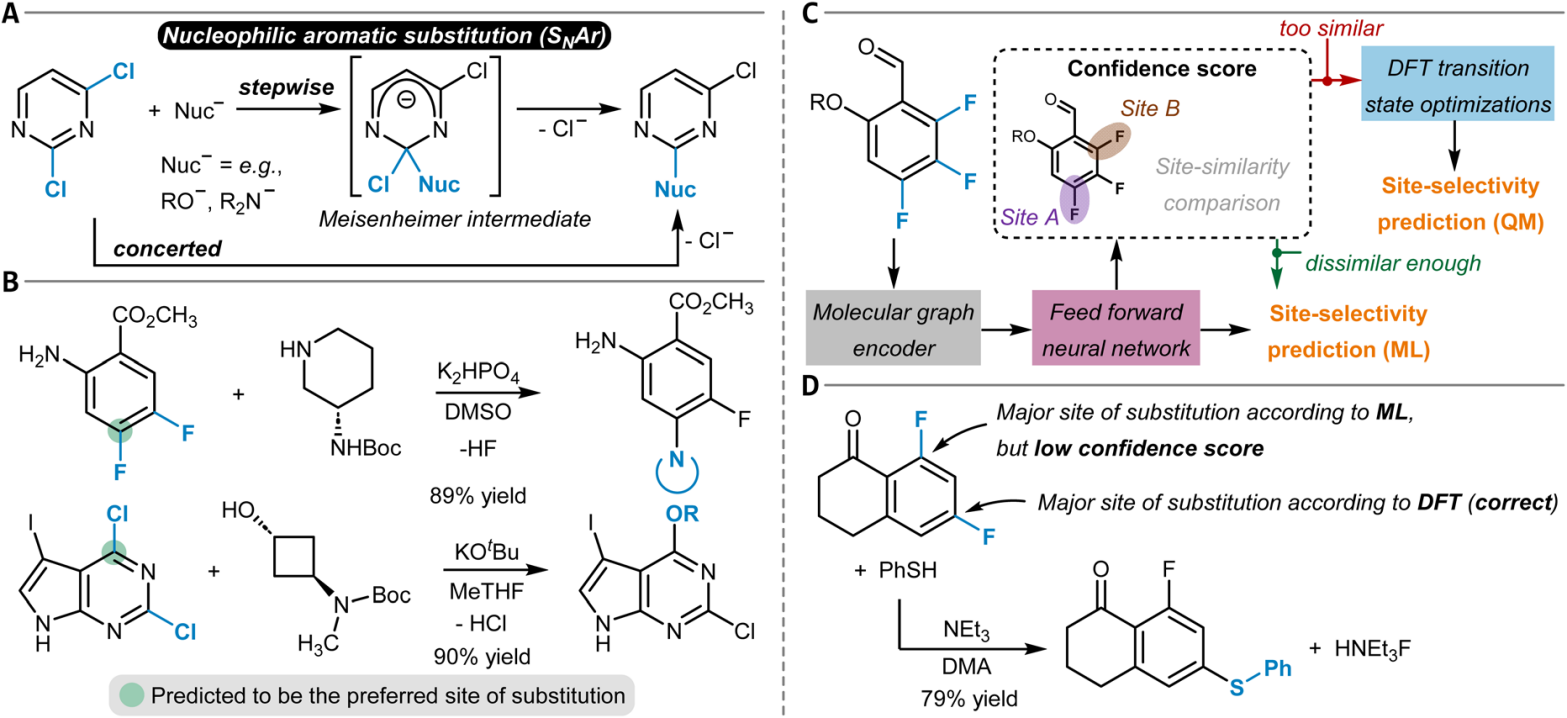
(A) Schematic reaction mechanism of a nucleophilic aromatic substitution reaction (S_N_Ar) either through a concerted or stepwise mechanism including a Meisenheimer intermediate. (B) Nucleophilic aromatic substitution reactions and their predicted site-selectivity from an MLR model. (C) S_N_Ar site-selectivity prediction workflow as developed by Guan *et al.* The reaction site-similarity provides a confidence score and is calculated as the distance in their latent space representations in the last layer of the GNN. (D) Application of the model shown in (C) to an S_N_Ar reaction of a difluoroarene with thiophenol as the nucleophile, which was incorrectly predicted by the ML part of the workflow, although with a low confidence score. This low confidence score triggered DFT optimization of the two individual transition states that corrected the initial erroneous prediction.

The chemical modification of aromatic C(sp^2^)–X groups, which is discussed in this section, can be achieved through two major reaction classes, both of great importance for organic synthesis: cross-coupling reactions and nucleophilic aromatic substitutions (S_N_Ar). Cross-coupling reactions typically install new C(sp^2^)–C(sp^2^) bonds through transition metal catalysis (Fig. 11A) and follow the general reaction scheme of oxidative addition, transmetalation, and reductive elimination.^272^ In S_N_Ar, the aromatic substrate and the nucleophile react directly with each other, either in a concerted or a stepwise mechanism, which involves a Meisenheimer intermediate^273^ to form a new C_aromatic_–N, O, or S bond in most instances (Fig. 12A).^274^ The oxidative addition step in cross-coupling reactions is often rate-limiting and thus site-selectivity determining. As oxidative addition (to a transition metal complex) can be viewed as a formal reduction of the organic substrate molecule, which is favored at the most electrophilic position, S_N_Ar and cross-coupling reactions follow similar selectivity trends.

### Cross-coupling reactions

Several review articles have been published on the site- and chemoselectivity question during cross-coupling reactions of polyhalogenated substrates.^275–278^ Quantum chemical calculations have been used to relate experimentally observed site-selectivities to the carbon–halogen BDE of the aryl halide and to the properties of its lowest-unoccupied molecular orbital (LUMO) through a distortion/interaction analysis.^279,280^ Ligand influences have also been discussed and experimentally investigated,^281,282^ and design of experiment (DoE) studies have been conducted for a specific reaction that is part of a kinase inhibitor synthesis.^283^

In general, the use of substrates with more than one leaving group can be synthetically more economical and straightforward than the sequential installation of each individual X group, given that the leaving groups can be targeted selectively during cross-coupling.^278^ A simple predictive tool is represented by Handy and Zhang's NMR chemical shift model (Fig. 11B).^284^ The order of functionalization of a polyhalogenated substrate is anticipated based on the ^1^H NMR chemical shift of the analogous non-halogenated molecules. The position with the larger chemical shift (more deshielded; more electrophilic) is predicted to react first. The model was successfully applied to thiophenes, furans, pyrroles, or pyridines (21 examples in total). This study is closely related to Kruszyk and others' work on S_E_Ar reactions^203^ (see above), and ML models for the prediction of NMR chemical shifts can help to facilitate this approach by providing fast descriptor access.^206,285^

Lu *et al.* developed an MLR model for relative rate constants of oxidative addition reactions to Pd(PCy_3_)_2_.^286^ The model was trained and tested (MAE of 2.3 kJ mol^−1^ (0.5 kcal mol^−1^)) with a dataset of 79 experimentally determined datapoints of (hetero)aryl chlorides, bromides, and triflates. As parameters, electrostatic potentials, *A*-values,^115^ intrinsic bond strength indices,^287^ and the p*K*_a_ value of the corresponding acid of the leaving group (*e.g.*, that of HBr in the case of aryl bromides) of the substrates were considered. The authors showed that their model can be used to predict the site-selectivity of Suzuki–Miyaura and Buchwald–Hartwig reactions of small polyhalogenated heteroaryl substrates correctly for 22 of the 24 tested molecules.

In two very recent studies, also GNNs were trained to predict the site-selectivity of cross-coupling reactions. Sakai *et al.* considered three elementary steps of the reaction (oxidative addition, substrate coordination to the transition metal, and reductive elimination).^288^ They used a large 914-dimensional one-hot-encoded atom feature input vector within their GNNs to predict the reaction probability between two atoms from the learned atom and bond embeddings, which they reported was possible with an overall accuracy of 97%.

Contrary to the work of Sakai *et al.*,^288^ Li, Liu, and others did not explicitly include organometallic chemistry in their model but focused instead on the two purely organic coupling partners to design a universal site-selectivity prediction model for cross-coupling reactions, including Buchwald–Hartwig, Suzuki–Miyaura, Stille, Sonogashira, Hiyama, Kumada, Negishi, and also Mizoroki–Heck transformations (see below).^289^ They compiled a dataset of 9734 reaction examples only including aryl substrate molecules with more than one potential site of cross-coupling. The models were supplied with both simple atom and bond descriptors (*e.g.*, atom symbols or valences, bond orders) and features from DFT (*e.g.*, Fukui coefficients, atomic partial charges). Importantly, the authors showed that the computationally expensive DFT features can be replaced with data from respective ML regression models without compromising model accuracy. Thereby, they follow the philosophy of ML-derived DFT descriptors as input to selectivity models as it was done by the groups of Green and Jensen^62^ (*cf.* the section on S_E_Ar). A checking algorithm for steric hindrance was also added to the overall model, resulting in the final Regio-MPNN tool (Table 1, entry 16). It achieved a prediction accuracy for the test set of 96% by probabilistically ranking a set of candidate products. Regio-MPNN was for example used to overrule the incorrect site-selectivity prediction of a general retrosynthesis planning program (Fig. 11C).

### Nucleophilic aromatic substitution reactions

Attempts at the computational prediction of the site-selectivity of S_N_Ar reactions have been made using several approaches. Peishoff and Jorgensen^290^ introduced the S_N_Ar reaction class to the CAMEO program in a similar fashion as it was done for the S_E_Ar reaction^191^ (see above). DFT studies have been conducted for specific systems,^291,292^ and C-DFT descriptors like Fukui indices,^293,294^ the local electron attachment energy,^158,295^ or the general-purpose reactivity indicator^296^ have been used to predict the site-selectivity of S_N_Ar reactions.

DFT calculations are also the basis for the so-called σ-complex approach,^189,190^ which uses the relative energy of the Meisenheimer complex (also denoted σ-complex, Fig. 12A) calculated for each potential position of substitution to predict the preferred reaction site (*cf.* the section on S_E_Ar reactions for a similar approach using relative Wheland intermediate stabilities instead^188^). The relative stabilities of the Meisenheimer complexes proved to be in good agreement with the experimental data and reproduced the observed site-selectivities. The method was applied to S_N_Ar reactions comprising both anionic and neutral nucleophiles, to substrates with different halide leaving groups, as well as to (per)fluorinated compounds,^297–299^ but it is limited to stepwise reactions that have a stable Meisenheimer intermediate.

In continuation of their work on oxidative addition to Pd(PCy_3_)_2_ (ref. 286) (see above), Lu and others developed an MLR model for the prediction of relative Gibbs free activation energies of S_N_Ar reactions trained with 74 experimentally determined datapoints. The electron affinity of the substrate as a global descriptor as well as the electrostatic potential at the reacting carbon atom and at respective *ortho* and *para*-positions as local descriptors proved sufficient to build an accurate model with an MAE of only 1.8 kJ mol^−1^ (0.4 kcal mol^−1^). The predictive tool was successfully applied in multiple case studies and showed high accuracy in predicting site-selectivity throughout – including several cases from medicinal chemistry research (Fig. 12B).^300^

Furthermore, hybrid approaches involving a combination of DFT modeling and ML beyond MLR have emerged as powerful solutions for S_N_Ar site-selectivity predictions. Jorner *et al.* developed a workflow capable of obtaining accurate values for absolute S_N_Ar reaction barriers with an MAE of around 3 kJ mol^−1^ (0.7 kcal mol^−1^) for a dataset of 443 experimentally determined free activation energies (Table 1, entry 17).^301^ Ground and transition states were calculated fully automatically at the DFT level with high robustness (success rate of above 98%), followed by the determination of site-specific features describing nucleophilicity, electrophilicity, steric, and dispersion interactions. Data on solvents was also included in the feature vector while the reaction temperature was excluded due to significant correlation with the prediction objective. Amongst various model architectures and feature combinations, optimal results were obtained with a Gaussian process regression model. Importantly, the model was also evaluated for its site-selectivity prediction capabilities and showed 86% accuracy on a respective subset of 66 reactions.

While Jorner's model provides highly accurate activation free energy predictions, even below the commonly accepted chemical accuracy level of 1 kcal mol^−1^ (4.184 kJ mol^−1^), it requires the optimization of transition structures for every possible site of substitution at the DFT level. This means the generation of the features for the actual Gaussian process ML model is quite time-consuming and potentially prone to errors. Within the Regio-MPNN model for cross-coupling reactions^289^ and the ml-QM-GNN reaction model^62^ (see above), DFT feature generation was substituted with much faster ML models that predict the respective DFT quantities. An alternative approach, which was followed by Guan and others for the S_N_Ar reaction, is to combine the accurate but slow DFT workflow with a separate and faster ML model and only explicitly calculate transition states in equivocal cases (Table 1, entry 18).^302^ Initially, a GNN makes site-selectivity predictions using DFT-calculated condensed Fukui indices for nucleophilic attack of the substrate molecule (electrophile) as atom features (Fig. 12C). Low-confidence predictions are then identified by comparing the learned site embeddings of the GNN. If they are found to be too similar, the explicit calculation of transition states for selectivity prediction with DFT is triggered. The method was trained and tested on a Pfizer internal dataset of around 3000 reactions as well as on 1760 public S_N_Ar reactions and the correct major product was found in 96.3% and 94.7% of the cases. Without explicit DFT analyses of respective transition states, the accuracies dropped to 91.9% and 90.8%, which demonstrates how ML and QM can work in accord to accelerate site-selectivity predictions with high accuracy (Fig. 12D). In the future, it could be attempted to substitute the DFT features of the GNN with data from respective ML models, which would speed up the entire workflow significantly. Also, the nucleophile could be included in the GNN model to account for potentially changing site-selectivity upon nucleophile variation, even though this does not frequently occur.^302^

## Functionalization reactions at multiple bonds

**Fig. 13:**
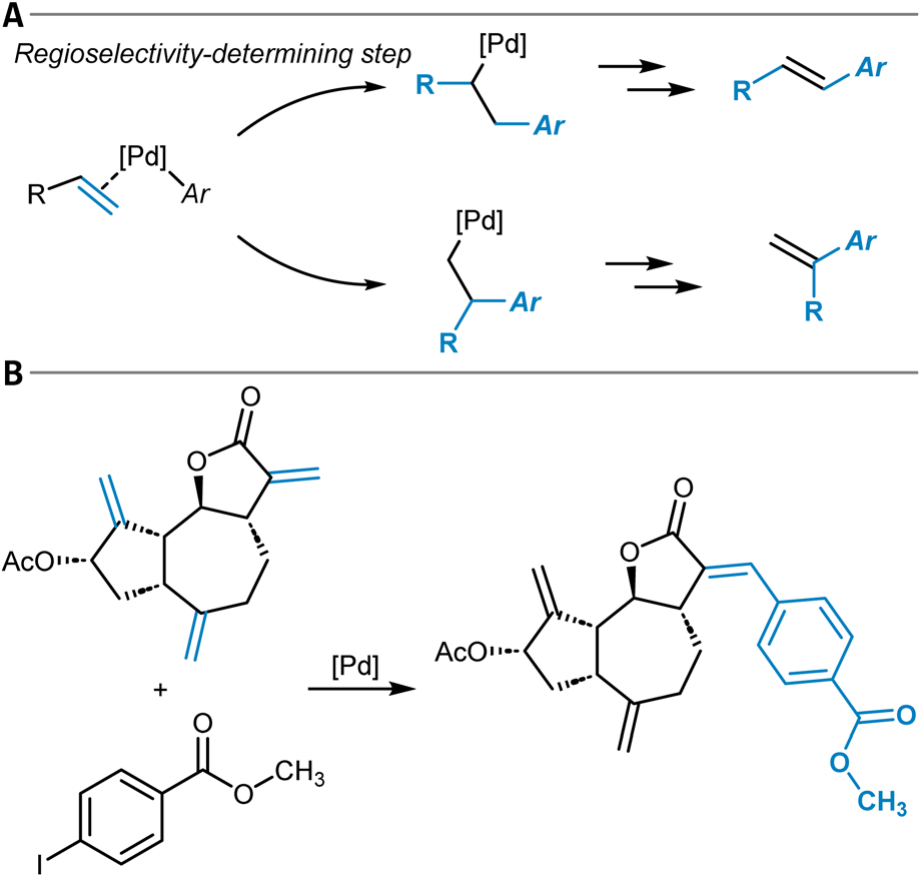
(A) Regioselectivity-determining alkene insertion step of the Mizoroki–Heck reaction leading to the two different regioisomers. (B) Reaction of a polyolefin that is part of Wang *et al.*'s Mizoroki–Heck dataset and that allows for the formation of site-isomeric products.

Chemical reactions at double and triple bonds come with the question on regioselectivity given that the unsaturated bond is unsymmetrically substituted and potentially given that the reaction partner is unsymmetrical as well (depending on the reaction type). In addition, site-selectivity can become of relevance when there is more than one reactive double or triple bond in the substrate (Fig. 13B). Often, stereoselectivity is of great relevance, too, which is however outside the scope of this paper. In principle, molecules with double or triple bonds can undergo substitution or (cyclo)addition reactions, and different computational models for their prediction have been developed. We start by discussing tools for the Mizoroki–Heck and hydroformylation reaction, followed by other addition reactions, including cycloadditions. Lastly, nucleophilic addition reactions to arynes are considered as a special case.

### Mizoroki–Heck reaction

The Mizoroki–Heck reaction is a powerful palladium-catalyzed method for the formation of new carbon–carbon bonds between olefin substrates and vinyl or (hetero)aryl building blocks and is closely related to the above-discussed cross-coupling reactions (Fig. 11A). Many experimental and quantum chemical studies have investigated the regioselectivity of the reaction, and its dependencies on factors like the electronic structure of the alkene substrate, the reaction conditions, or the chosen ligand.^303,304^ Deeth *et al.* developed a selectivity index with an electrostatic and orbital interaction component, quantum chemically calculated from the reactive intermediate prior to the regioselectivity-determining migratory insertion step (Fig. 13A).^305^ Selectivity scales for a set of common substituents were reported for the neutral and cationic reaction path,^306^ which can be used to gauge the directing influences of a given group to enforce either one of the two possible regioisomers. Another computational study also considered the steric influences of the ligand on the regioselectivity.^307^

To automate quantum computational calculations of Mizoroki–Heck reactions, the Jensen group developed a workflow for the prediction of the regioselectivity of intermolecular reactions at a mixed DFT and SQM level of theory – also considering both, the neutral and cationic reaction path (Table 1, entry 19).^242^ Their model showed moderate accuracy (63% and 29% for predicting the two possible regioisomers through the neutral and cationic pathway, respectively) on a large dataset of 3342 reactions extracted from Reaxys, which was discussed in the context of the above-mentioned multidimensionality of factors influencing regioselectivity. This illustrates the challenges associated with going from a small set of model systems to a broad variety of real-world examples in the context of mechanistically intricate transformations like the Mizoroki–Heck reaction.

An alternative to the automated quantum chemistry approach is a more data-driven strategy, in which the diversity in regioselectivity is directly inferred from reaction data without explicit mechanistic assumptions during modeling. In this vein, Wang *et al.* trained a transformer-based ML model for the prediction of product SMILES strings of Mizoroki–Heck reactions from the reactants by making use of a transfer learning strategy.^308^ Initial training with a general reaction database was followed by fine-tuning with a set of 9959 Mizoroki–Heck reactions. The model achieved high accuracies for both inter- and intramolecular reactions (95% for the entire test set). Importantly, they also investigated the performance of their model for 375 polyolefinic and 408 polyhalogenated substrates and found prediction accuracies of 85% and 92%, respectively. Such cases include the possibility for the concomitant formation of isomers due to site- and regioselectivity (Fig. 13B).

The aforementioned Regio-MPNN^289^ (*cf.* the section on cross-coupling reactions) also covers the Mizoroki–Heck reaction, and it can be used to make site-selectivity predictions for both polyolefin and polyhalogenated molecules. The possibility of different regioisomers at a given double bond site is considered in certain cases. Future research efforts could focus on a further generalization of selectivity models for the Mizoroki–Heck reaction which includes careful testing for both selectivity types.^309^ Furthermore, the sole focus on the reactants could be widened to also include more details on reaction conditions, which can influence selectivity.

### Hydroformylation reaction

**Fig. 14:**
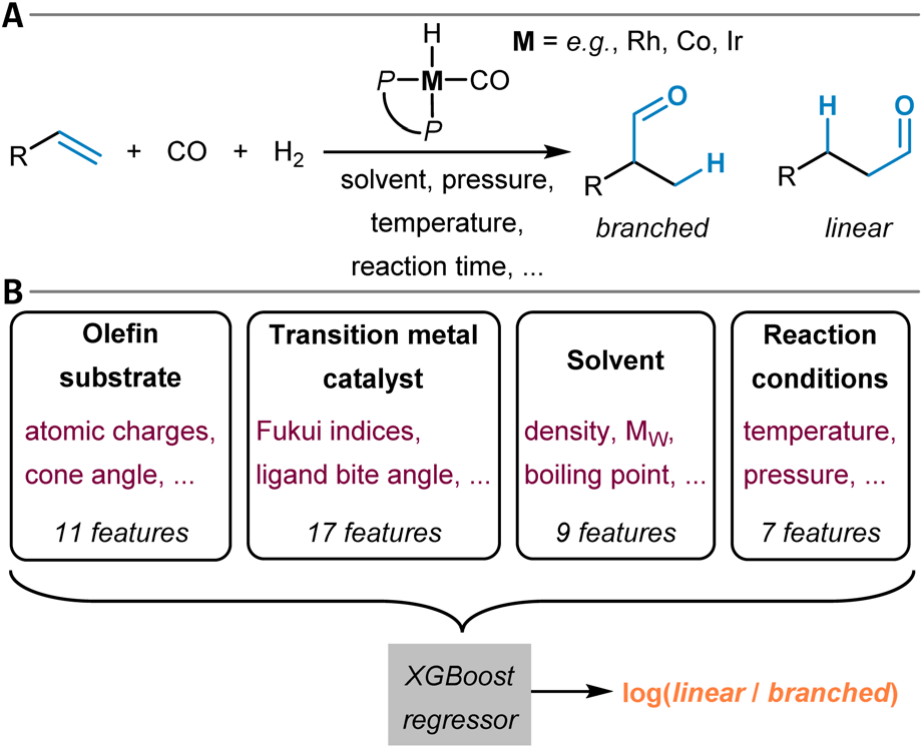
(A) General reaction scheme of a hydroformylation reaction of a terminal olefin catalyzed by a phosphine-ligated transition metal central atom and the two possible regioisomeric reaction products. (B) Schematic representation of Wang *et al.*'s hydroformylation regioselectivity model for terminal olefin substrates.

The hydroformylation reaction is the addition of H_2_ and CO to a double or triple bond, typically catalyzed by a cobalt or rhodium catalyst, to give aldehyde products (Fig. 14A). Given the significant relevance of this reaction for industry, several contributions have been made to rationalize and predict its regioselectivity, including efforts with QM simulations.^310–313^ The hydroformylation of double bonds is mechanistically related to the Mizoroki–Heck reaction in the sense that it includes the insertion of the olefin into a transition metal-element bond as the regioselectivity-determining step (Fig. 13A). This is either a transition metal–hydrogen bond in the case of hydroformylation or a transition metal–carbon bond for the Mizoroki–Heck reaction. Therefore, similar approaches for modeling the regioselectivity of these two reactions have been pursued. Sigman and coworkers, for example, found the difference in ^13^C NMR chemical shift between the two alkene carbon atoms a good descriptor to predict the regioisomer ratio of oxidative Heck reactions.^314^ Later, similar trends were found for the hydroformylation reaction.^315^ Recently, Linnebank and others combined the ^13^C NMR chemical shift difference with the intensity of the stretching vibration of the C=C double bond within a linear regression model and obtained an improved correlation (*R*^2^ = 0.86 *vs.* 0.74) for their set of 41 terminal olefins which were subjected to a rhodium-catalyzed hydroformylation.^316^ A related reaction in this context is a rhodium-catalyzed arene annulation to form lactams, which includes a directed oxidative addition of a C_aromatic_–H bond (see above) followed by an olefin insertion into the Rh–C bond as the regioselectivity-determining step. MLR models were built to identify ligands that result in high regioselectivity as probed with a model reaction.^317^

Coming back to hydroformylation, Wodrich *et al.* used molecular volcano plots constructed from linear free energy relationships to computationally search rhodium catalysts with diphosphine ligands for the hydroformylation of isobutene.^318^ Volcano plots relate a selected relative free energy of an intermediate in a catalytic cycle (thermodynamic descriptor) to the free energies of all other intermediates for a set of different catalysts which results in a volcano-like diagram.^319^ In their study, the authors showed that the activation Gibbs free energy for the critical insertion step of the olefin into the Rh–H bond correlates well with the Gibbs free energy of the following intermediate (Fig. 13A), which allowed them to identify ligands that result in the selective formation of either of the two regioisomers.

Similar to the Mizoroki–Heck reaction, the regioselectivity of hydroformylations is influenced by the reaction conditions – perhaps even to a greater extent. Therefore, Wang and others manually extracted data on reaction conditions like solvent, pressure, or reaction time when they compiled a database of 1167 literature-known hydroformylation reactions of terminal olefins catalyzed by diphosphine-ligated transition metals.^320^ Features for the olefin and the transition metal catalyst were obtained from QM simulations and were combined with the general reaction information to predict the regioisomer ratio with the XGBoost algorithm (Fig. 14B and Table 1, entry 20). SHAP values^321^ were used to identify the atomic partial charges of the olefin carbon atoms and the respective cone angle as most influential on the model's predictions. The authors also trained substrate-specific models for the most prevalent olefins, oct-1-ene and styrene, and observed improved performance compared to the general model.^322^

### Further addition reactions to double bonds

Beyond hydroformylation, double bonds can undergo a wide range of other addition reactions following, for example, radical or electrophilic reaction mechanisms; potentially catalyzed by transition metal complexes (see below for cycloaddition reactions). Their regioselectivity has been the subject of a plethora of quantum chemical studies.^323–325^ Well-known is Markovnikov's rule for the addition of hydrogen halides to double bonds, which assigns the added hydrogen atom to the least substituted position of the alkene; although this rule does not apply to radical reactions.^326,327^ The empirical Markovnikov rule was investigated in several quantum chemical studies and was, for instance, related to different Fukui indices.^328–331^ In the early 1990s, Elrod *et al.* were able to reproduce the rule with small neural networks trained to predict the regioselectivity of the addition reaction of hydrogen halides to double bonds.^332^ It also emerged from an ML-driven reaction network study in which products of organic reactions along with their reaction pathway were predicted.^333^

**Fig. 15:**
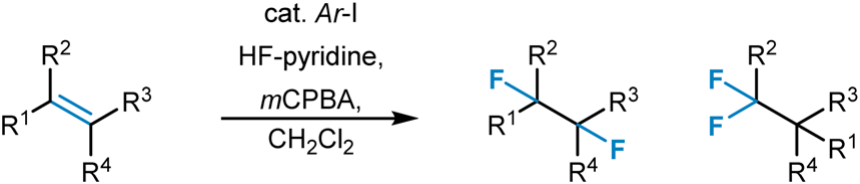
Aryl iodide-catalyzed difluorination of alkenes and the two possible isomeric reaction products.

The Markovnikov rule is a general guideline for reactivity prediction, though regioselectivity prediction tools for specific addition reactions to double bonds are rare. One example came from the Sunoj group. They trained neural network models for the prediction of the difluorination of olefins with a hypervalent iodine-based catalytic system.^334^ The objective was to distinguish between vicinal and geminal difluorination, with the latter involving a 1,2-shift of one of the double bond's substituents (Fig. 15). Features such as atomic partial charges, Fukui coefficients, or NMR chemical shifts, as well as structural features for 66 datapoints were obtained from DFT calculations resulting in a model with 90% classification accuracy.

### Cycloaddition reactions

Another very important class of addition reactions to double but also to triple bonds is cycloaddition, which is used to construct new ring structures. Due to their special and usually concerted reaction mechanism, they were heavily investigated in quantum chemical studies, not least on regioselectivity.^335,336^ Various C-DFT descriptors, like Fukui indices or local electrophilicity and softness values as well as frontier molecular orbital theory, were used to predict the regioselectivity of Diels–Alder and 1,3-dipolar reactions, which are two prominent classes of cycloadditions.^57,337,338^ Early rule-based synthesis prediction tools like CAMEO^339,340^ and EROS^341^ implemented some of these findings into automated computational routines, which achieved prediction accuracies of over 90% based on MLR and which were successfully applied to a variety of real-world examples.

Later, several research efforts focused on predicting reaction barriers of cycloadditions, especially of Diels–Alder reactions, beyond explicit quantum chemical simulation by making use of ML. Different featurization techniques were deployed, including structural information on the reactants, data on reaction conditions^342^ as well as quantum chemically derived descriptors from SQM calculations^343^ or QTAIM analyses.^344^ Model architectures like support vector regression, tree-based methods, and neural networks were trained either to predict experimentally determined or DFT ground truth data. In principle, such reaction barrier models can be used to also predict reaction selectivities, including regio- and site-selectivity, which could be the subject of future research efforts.

**Fig. 16:**
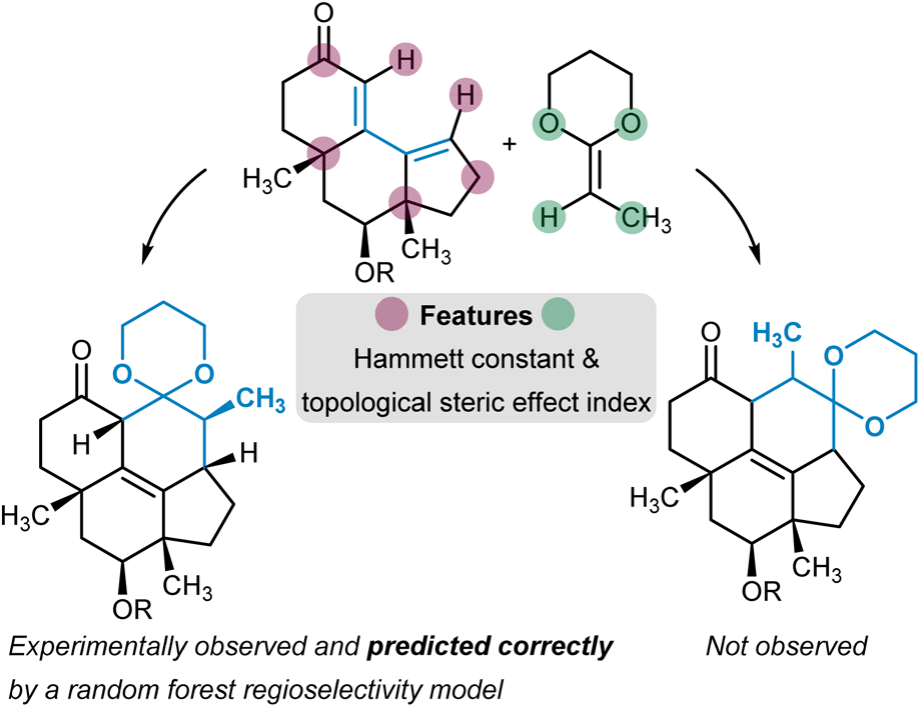
Diels–Alder reaction en route to the total synthesis of rippertenol with the two possible regioisomers (*cf.* ref. 348). The experimentally observed regioselectivity was correctly predicted by an RF model based on the Hammett constants and the topological steric effect indices of the dienophile's and diene's substituents.

The first ML models for the dedicated data-driven prediction of site- and regioselectivity of Diels–Alder reactions were published by the Grzybowski group in 2019 (ref. 345) followed by a recent paper from Wiest and coworkers.^346^ The initial contribution reported on RF classifiers for regio- and site-selectivity based on a dataset of 6355 intermolecular reactions, which was possible with 93.6 and 91.3% accuracy. For the featurization of the substrate molecules, all substituents of the reacting diene and dienophile were described by their Hammett constant (electronic influence) and topological steric effect index^347^ (TSEI, steric influence) (Fig. 16).^348^ The authors investigated the importance of these physically meaningful descriptors and ascribed a higher degree of generalizability to the resulting models compared to models trained with topological fingerprint features, which are not rooted in physics.^349^

The Wiest group worked with a similar dataset (9537 datapoints) but also included intramolecular Diels–Alder reactions.^346^ They trained a graph-based Non-autoregressive Electron Redistribution Framework (NERF)^350^ with their dataset and obtained prediction accuracies of over 90% (Table 1, entry 21). Importantly, readily available node attributes such as atom type and formal charge proved sufficient, thus excluding the need for more expensive features derived from quantum chemistry. This demonstrates that GNNs, especially architectures like NERF, which is inspired by the electron redistribution picture for the mechanism of chemical reactions (arrow pushing), are capable of learning molecular representations suitable for highly accurate predictions. This is possible without the supply with physics-derived descriptors as long as there is enough training data. In contrast, algorithms like decision tree-based methods that do not perform representation learning benefit from physically motivated features as described above.^345^

**Fig. 17:**
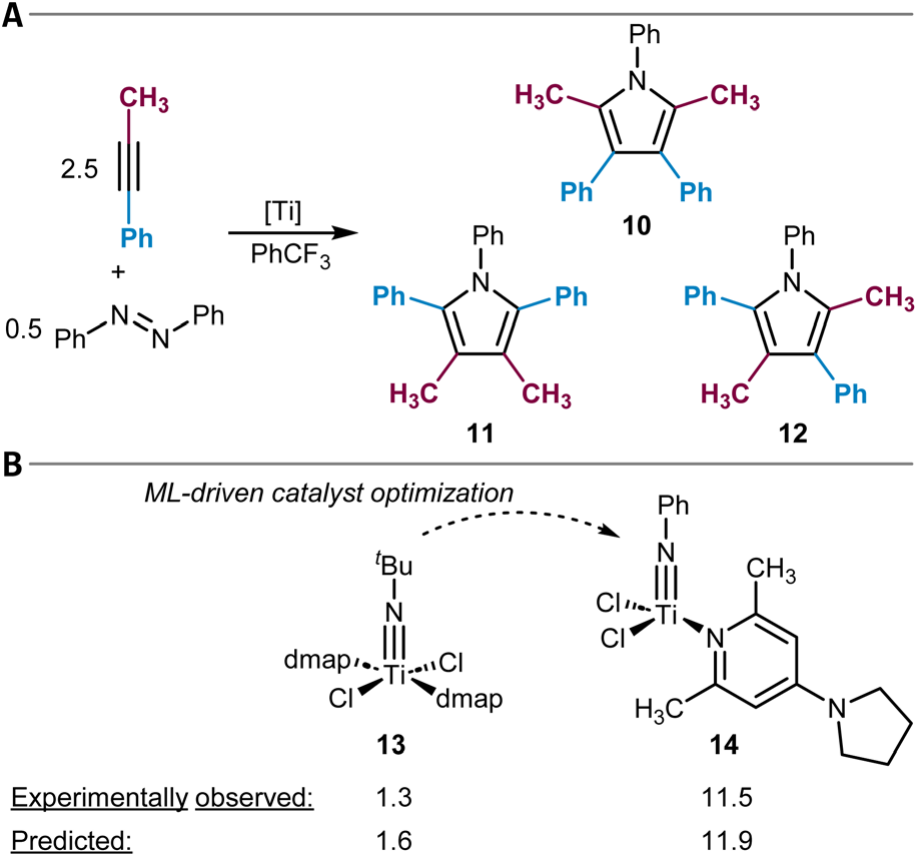
(A) Titanium-catalyzed [2 + 2 + 1]-cycloaddition of 1-phenyl-1-propyne and azobenzene to give pyrrole derivatives 10 to 12. (B) Catalyst optimization to maximize the production of the desired regioisomer 10. The reported selectivities refer to 10/(11 + 12).

The most important cycloaddition reactions of triple bonds are the azide–alkyne reactions. Transition metal catalysis with copper or ruthenium renders them highly regioselective due to complex reaction mechanisms, which were studied in great detail with quantum chemical calculations.^351,352^ Predictive tools for reaction feasibility are therefore more relevant in this context compared to selectivity models.^353^ Instead, data-driven regioselectivity studies focus on more specialized cycloaddition reactions of alkynes in which regioselectivity is less clear. For instance, iterative supervised principal component analysis was used to optimize titanium catalysts for the [2 + 2 + 1]-cycloaddition between alkynes and azobenzene to yield pyrroles (Fig. 17).^354^ Descriptors for the electronic and steric features of the Ti-complexes were obtained from DFT calculations, and the final model successfully identified compound 14 as a highly regioselective catalyst.

### Reactions of arynes

**Fig. 18:**
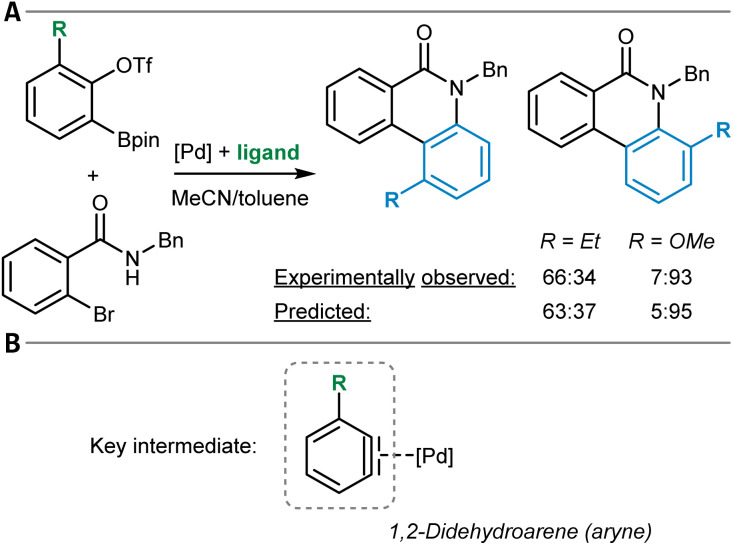
(A) Palladium-catalyzed annulation of *ortho*-borylaryl triflates and the two possible reaction products with experimentally observed regioselectivities and the respective predictions from a linear model. The Hammett and Charton parameters of the R substituent and the cone angle of the applied ligand at palladium were used to predict regioselectivity. (B) The key intermediate of the reaction shown in (A), which is the palladium complex of the *in situ*-generated aryne.

A special class of alkynes are 1,2-didehydro(hetero)arenes commonly referred to as (hetero)arynes (see Fig. 18B for a palladium complex of an aryne). They are generated *in situ* from suitable precursors and allow for a diverse functionalization of the parent (hetero)arenes due to their ring strain-induced high reactivity.^355^ The distortion/interaction model was found to provide a quantitative metric to predict the regioselectivity of such reactions based on DFT calculations^356–359^ – which is in fact also the case for the just discussed cycloaddition reactions to alkynes.^360^ Automated distortion/interaction analyses are possible with autoDIAS which offers a simple and systematic way to generate the required molecular structures.^361^ Also, steric influences were discovered to compete with the pure distortion model for certain silylarynes.^362^ Beyond the distortion/interaction model, the regioselectivity of aryne reactions was rationalized with frontier molecular orbital considerations^363^ or the orbital electronegativity descriptor.^364^ The latter approach reported by Mirzaei and Khosravi does not require quantum chemical calculations and provided qualitatively correct predictions for 29 of the 30 tested (hetero)arynes. The carbon atom with the lower π-orbital electronegativity as calculated with MarvinSketch indicates the preferred position of nucleophilic addition.^364^

Aryne functionalization reactions within the coordination sphere of transition metal complexes often cannot accurately be described with the distortion/interaction model as the regioselectivity in these reactions is influenced by additional factors. Plasek *et al.* investigated this phenomenon for a series of 43 palladium-catalyzed aryne annulations.^365^ They developed an MLR model for the prediction of experimentally determined ΔΔ*G*^‡^ values based on parameters for the electronics and sterics of the aryne substrates (Hammett and Charton parameter^366^) and also included the cone angle of the ligand at palladium as a feature. They demonstrated that their model can accurately extrapolate to a ligand excluded from training with an MAE of only 0.6 kJ mol^−1^ (0.1 kcal mol^−1^, Fig. 18).

## Conclusion and outlook

This article gives an overview of the currently available computational tools for the prediction of regio- and site-selectivity of organic reactions. The main focus was put on functionalizations of C–H groups due to their omnipresence in organic molecules, which makes the development of selective reactions and corresponding predictive tools particularly challenging. Substitution reactions at C–X moieties as well as reactions at double and triple bonds were covered as well.

In the past, regio- and site-selectivity were most commonly modeled with quantum chemical simulations, either through explicit mechanistic considerations or the analysis of substrate molecule descriptors, for example, obtained from conceptual DFT. In the last decade, the research boundaries were increasingly pushed toward the extension of these often accurate yet slow and potentially error-prone approaches with more powerful ML models trained on experimental selectivity data. This is done to provide faster predictive tools that can be easily applied by practitioners. Many of these tools are publicly available and sometimes even come with an online graphical user interface (Table 1).

For smaller datasets of specific, mostly transition metal-catalyzed reactions, multiple linear regression was frequently applied, often in combination with specially developed features obtained from DFT. This resulted in easily interpretable models that were, for example, used for the rationalization or optimization of catalytic systems. For more common reactions like aromatic substitutions with thousands of datapoints available, more intricate ML models like graph neural networks have been trained. While graph neural networks can make use of DFT data as input features to learn more accurate molecular representations, especially in regimes of lower data availability, it was also explored how DFT-calculated features can be replaced with ML-predicted features, resulting in even faster site- and regioselectivity prediction. In the case of the deep learning-based tools, less focus has so far been put on model interpretability, but rather on end-to-end solutions ready to be deployed to respective use cases including the application to large compound libraries.

Throughout the paper, we have mentioned several opportunities for potential future research. These include the development of regio- and site-selectivity prediction tools for new reactions. Also, new or updated models could be supplied with data on reaction conditions in cases in which a significant influence on selectivity is expected.^188^ At the moment, the ample inclusion of condition data into feature vectors is less common and its potential could be investigated. Another conceivable future research opportunity could be to extend regio- and site-selectivity models to closely related areas like chemoselectivity. The prediction of reaction feasibility is also of major importance,^367^ which can be achieved through dedicated feasibility models or combined feasibility and selectivity tools. Increasing the generalization of the tools to a broader set of reaction classes, for instance, through transfer learning, could allow the building of accurate ML models for reactions with only limited amounts of available data.^201^ All these opportunities should go along with the widespread application and thorough benchmarking of the available tools, which at the same time can result in the generation of new data for model training and evaluation. For software developers, this means providing easy-to-use and well-explained implementations of their models – optimally through graphical user interfaces.^368^ For synthetic chemists, this means utilizing the available tools and reporting their usage,^369^ as well as documenting reaction data in a format suitable for ML – including the critically needed “negative data”.^370,371^ Diverse and high-quality experimental and also computational datasets and their in-depth analysis are the essential foundation for the advancement of site- and regioselectivity ML models.^372^

**Fig. 19:**
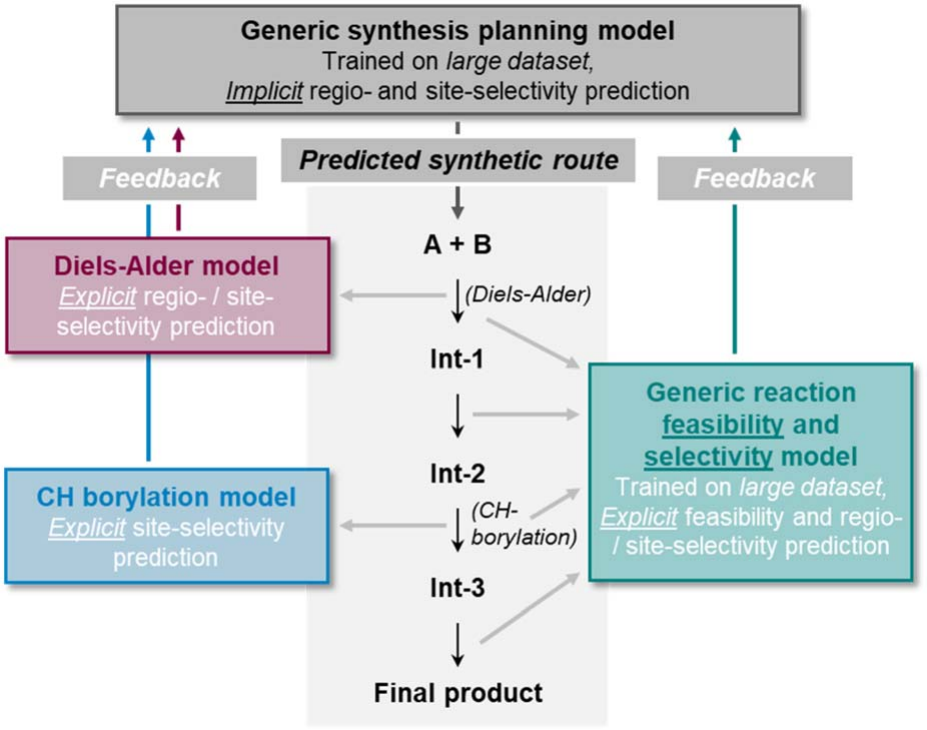
Schematic representation of how generic synthesis planning software (including retrosynthesis tools) can work in cooperation with explicit regio- and site-selectivity models for the overall improved prediction of synthetic pathways (left part). In the future, increasing generalization of reaction selectivity but also feasibility tools could be sought to evaluate each predicted synthetic step (right part).

In the future, we believe that regio- and site-selectivity prediction tools will have an important role to play and will be available to end users through synthesis planning software.^373^ Retrosynthesis algorithms will suggest plausible routes and general-purpose forward prediction tools can give a preliminary assessment of their feasibility. A more stringent evaluation in terms of selectivity can be done with specialized tools (Fig. 19). Future developments will increasingly work toward the generalization of these models and design them to also handle reaction feasibility. These tools would be able to make predictions with different speed and accuracy depending on the context, for example, in drug discovery or in process chemistry, where timelines and acceptable levels of yields and purities differ. Such tools can be used by humans to quality check suggested routes, or plausibly by autonomous artificial intelligence agents that operate with several synthesis planning tools at their disposal to deliver higher-quality routes to the human decision-maker.^374,375^

## Data availability

No primary research results, software or code have been included and no new data were generated or analysed as part of this review.

## Author contributions

L. M. S. conducted the literature search and wrote the manuscript with M. A., M. J. J., M. K., and K. J. All authors contributed to finalizing the paper and agreed to publish the submitted content. The AI tools DALL E 3 (generation of the stylized person in the TOC figure), ChatGPT-4o (rephrasing of individual sentences), and Grammarly (free version; grammar, wording, and punctuation review) were used.

## Conflicts of interest

There are no conflicts to declare.
